# ADP-ribosyltransferases Parp1 and Parp7 safeguard pluripotency of ES cells

**DOI:** 10.1093/nar/gku591

**Published:** 2014-07-17

**Authors:** Stephen J. Roper, Stephanie Chrysanthou, Claire E. Senner, Arnold Sienerth, Stefano Gnan, Alexander Murray, Mitsuko Masutani, Paulina Latos, Myriam Hemberger

**Affiliations:** 1 Epigenetics Programme, The Babraham Institute, Babraham Research Campus, Cambridge CB22 3AT, UK; 2 Centre for Trophoblast Research, University of Cambridge, Downing Street, Cambridge CB2 3EG, UK; 3 Division of Genome Stability Research, National Cancer Center Research Institute, 5–1–1 Tsukiji, Chuo-ku, Tokyo 104–0045, Japan

## Abstract

Embryonic stem (ES) cells are in a dynamic equilibrium of distinct functional states, characterized by the heterogeneous expression of critical pluripotency factors and regulated by a spectrum of reversible histone modifications. Maintenance of this equilibrium is a hallmark of pluripotency. Here we find that the ADP-ribosyltransferases Parp1 and Parp7 play a critical role in safeguarding this state by occupying key pluripotency genes, notably *Nanog*, *Pou5f1*, *Sox2*, *Stella*, *Tet1* and *Zfp42*, thereby protecting them from progressive epigenetic repression. In the absence of either Parp1 or Parp7, or upon inhibition of the ADP-ribosylating activity, ES cells exhibit a decrease in ground state pluripotency as they cannot maintain the typical heterogeneity characteristic of the metastable state. As a consequence, they display a higher propensity to differentiate. These findings place Parp1 and Parp7 at the genetic-epigenetic interface of pluripotency networks, fine-tuning the transcriptional heterogeneity and thereby determining the developmental plasticity of ES cells.

## INTRODUCTION

Regulation of stem cell self-renewal and differentiation is underpinned by epigenetic modifications encompassing covalent changes to the DNA itself as well as histone modifications. Together, these marks influence chromatin organisation and accessibility. Perhaps the least studied of the various histone modifications is ADP-ribosylation. ADP-ribosylation is a post-translational protein modification conferred by poly-ADP-ribose polymerases (Parp's) that catalyse the transfer of ADP-ribose moieties to acceptor proteins. Despite their name it has emerged that some of these Parp's catalyse the addition of only a single ADP-ribose unit (mono-ADP-ribosylation), while others add multiple moieties to form linear or branched poly-ADP-ribose (PAR) chains (1–3). Addition of PAR chains imposes structural constraints and introduces negative charges to acceptor proteins, thereby altering their interactions with other proteins and with DNA (4). Amongst the target proteins, histones represent very prominent acceptors of ADP-ribosylation, which is most commonly associated with the relaxation of chromatin structure (5–7). Although in general, Parp's are best known as key players in DNA repair, they have a broad repertoire of functions and their contributions to chromatin remodelling and transcriptional regulation have gained significant attention recently (8).

The precise mechanisms how Parp's, and in particular the most comprehensively studied family member Parp1 (also known as Artd1 (2)), act in these various processes can be on multiple levels; as the protein itself, as auto-modified protein or as the result of modifying other target proteins. This complexity often complicates the analyses and interpretation of data. In addition to modifying histones, Parp1 can displace the linker histone H1 at gene promoters to enforce a decondensed, open chromatin organisation (9). Concomitantly, Parp1 prevents demethylation of H3K4me3 through the PARylation, inhibition and exclusion of the histone demethylase Kdm5b (10). Collectively, these functions help maintain an active chromatin configuration at target loci, for example at the promoter of the DNA methyltransferase *Dnmt1*, such that Parp1 seems to directly regulate the transcriptional activity of this epigenetic modifier (11). However, in a seemingly opposing function, Parp1 and Parp2 (also known as Artd2) are also important for ensuring the integrity of constitutive and facultative heterochromatin (4,12–14). It has been speculated that these antagonistic roles in both chromatin decondensation and chromatin compaction may be attributable to the precise levels of Parp1 activation (11).

Overlaying these direct effects on chromatin configuration, a number of epigenetic modifiers, including Kdm5b, the heterochromatin protein Hp1α (15), the nucleosome remodelling ATPase Iswi (16) and the transcription factor Ctcf (17), are themselves targets of PARylation. Importantly, auto-modified Parp1 can directly interact with the Dnmt1 protein thereby reducing its DNA methyltransferase activity (18). Both Parp1 and Dnmt1 have been isolated from a replication fork complex together with the ubiquitin-like protein Np95 (also known as Uhrf1), the histone methyltransferase G9a (Ehmt2) and Pcna (19).

It is likely that at least some of these chromatin-modifying functions underlie the role for Parp1 in somatic cell reprogramming towards induced pluripotent stem (iPS) cells. Thus, Parp1 has been reported as an important factor specifically for the early stages of iPS cell reprogramming, preceding the actual transcription of pluripotency genes such as *Esrrb* and *Nanog*, by affecting DNA methylation, enrichment of active chromatin marks and Oct4 binding to these sites (20). Interestingly, Parp1 can replace Klf4 or c-Myc as reprogramming factors, a role that may—at least partially—be explained by Parp1 representing a direct transcriptional target of c-Myc (21). Finally, there is mounting evidence of an interaction between Parp1 and Sox2 through which an impact on reprogramming efficiency may be exerted, even though the precise nature of this association is not entirely clear yet. Thus, it has been reported that Parp1 may directly bind to and PARylate Sox2, thereby altering its regulatory role at the *Fgf4* enhancer resulting in up-regulation of *Fgf4* transcriptional activity, which may be beneficial for reprogramming efficiency (22,23). By pulling down Sox2-binding proteins, Lai *et al.* also identified Parp1 as a Sox2 complex component. However, they report that this interaction occurs with auto-PARylated Parp1, is enhanced by Fgf4 signalling and prevents Sox2 from binding to cognate Oct/Sox motif-containing enhancers (24). These examples highlight the complexity of Parp1's functions introduced by the difficulty in discriminating covalent from non-covalent associations with PAR chains and the precise effects on chromatin organisation conferred by Parp1 in different contexts. While Parp1 has been in the focus of many of these studies, it has remained largely unknown whether or not other members of the Parp superfamily also contribute to the regulation of nuclear architecture in stem cells and reprogramming.

Of the multiple facets of Parp biology, our interest stems from the observation that *Parp1*-deficient embryonic stem (ES) cells, unlike their wildtype counterparts, are capable of differentiating into derivatives of the extraembryonic trophoblast lineage. This phenotype was evident *in vitro* and even more obvious *in vivo* in ES cell-derived teratocarcinoma-like tumours that developed massive haemorrhagic areas as a consequence of trophoblast giant cell differentiation (25,26). Trophoblast differentiation potential of ES cells is remarkable because in the mouse, ES cells are normally excluded from contributing to this extraembryonic placental lineage (27). Differentiation into the trophoblast lineage can only be achieved by manipulation of ES cells to either lower the established epigenetic barriers, for example by hypomethylation or by interfering with the H3K9 methylation machinery; or by modulating critical transcription factors such as overexpression of *Cdx2* or knockdown of *Pou5f1* (encoding the transcription factor Oct4) or *Nanog* (28–35). We thus set out to determine whether the ‘trans'differentiation ability of *Parp1*^–/–^ ES cells, and ADP-ribosylation in general, is linked to any of these mechanisms and thereby contributes to determining stem cell identity. Our results demonstrate that Parp1 is complemented by Parp7 to restrict lineage fate by maintaining an active epigenetic state at key pluripotency factors. These findings implicate both activities as core components of the pluripotency network and as determinants of the developmental plasticity of stem cells.

## MATERIALS AND METHODS

### Cell culture

Wildtype ES cell lines used were J1, E14tg2a and B6. *Parp1^–^*^/–^ 210–58 ES cells were described previously and are on a J1 background (36). RRG177 ES cells gene-trapped at the *Parp7* locus (Bay Genomics) were obtained from the MMRRC, University of California, Davis (USA) and were on an E14tg2a background. *Parp7^–^*^/–^ ES cells were generated by culturing RRG177 (*Parp7*^+/*–*^) ES cells in ES cell medium supplemented with 2 mg/ml G418 for 16 days. The gene trap insertion was mapped to intron 3 of *Parp7*, upstream of the Parp catalytic domain. ES cell culture was performed under standard conditions containing 15% foetal bovine serum and 10^3^U LIF (37). For generation of *Rex1*-GFP knock-in cell lines, a destabilized GFP (GFPd2) was inserted into the *Rex1* (also known as *Zfp42*) locus using a construct previously described (38). For Parp inhibition experiments, the broad-spectrum Parp inhibitor PJ34 (Sigma) was added at a final concentration of 5 μM.

Trophoblast stem (TS) cell lines used were TS-GFP and TS-Rs26, cultured in standard TS medium (39) containing 20% foetal bovine serum, 25 ng/ml bFGF (Sigma) and 1 μg/ml Heparin, with 70% of the medium pre-conditioned on embryonic feeder cells. In transdifferentiation experiments, ES cells were plated in TS medium on gelatin-coated dishes in the absence of a feeder-cell layer.

For embryoid body differentiation, 5 × 10^3^ ES cells were plated in hanging drop culture for 3 days in medium with reduced foetal bovine serum content (10%) and without LIF, and then transferred onto non-adherent dishes cultured on a rocking platform for another 5 days, with media changes every 2–3 days. For retinoic acid differentiation experiments, cells were plated on normal tissue culture dishes in medium with reduced foetal bovine serum content (10%) and without LIF in the presence of 0.16 μM retinoic acid (Sigma).

For expression of FLAG- and His-tagged Parp1 and Parp7, full-length sequence-verified open reading frames were cloned into the pIRES-hrGFP-1a (Stratagene), pcDNA3.1 (Invitrogen) and pCAG-IRES-Zeocin vectors. For shRNA experiments, three constructs were tested for knockdown efficiency and the best two chosen for the experiments displayed. Vectors were transfected into ES cells with Lipofectamine 2000 reagent (Invitrogen) according to manufacturer's instructions. Immunolocalisation studies were performed in transiently transfected COS-7 cells.

### Immunofluorescence staining

Cells were grown on glass cover slips, fixed with 4% PFA for 10 min and permeabilized with 0.1% Triton X-100 in phosphate buffered saline (PBS). Antibodies used were: Cdx2 (Biogenex CDX2–88) at 1:400, Elf5 (Santa Cruz Biotechnology N-20 sc-9645) 1:200, His-probe (Santa Cruz Biotechnology) 1:100, FLAG (M2, Sigma F1804) 1:400, Nanog (Abcam ab19857) 1:400, Oct4 (Abcam ab19857) 1:400, Parp1 (Santa Cruz Biotechnology sc-74469x 1:200 and Abcam ab18376 1:100, both giving identical results), Parp7 (Abcam ab170817) 1:100 and Stella (Abcam ab19878) 1:100. Secondary antibodies were Alexa fluorophores (Molecular Probes) at 1:400, and DNA was visualized with DAPI or bis-benzimide. Photographs were taken on an Olympus BX41 epifluorescence microscope and a Zeiss 510 Meta confocal microscope, and analysed with Volocity software (Improvision). Cells (*n* > 1000) were classified as positive or negative for each factor analysed and data compared using a Chi-squared test (*P < 0.05, **P < 0.01, ***P < 0.001).

### Fluorescence activated cell sorting

ES cells stained for Cdx2 were fixed in suspension with 1% PFA for 10 min, permeabilized in 0.2% Triton X-100 in PBS for 10 min and then blocked in 0.5% BSA, 0.1% Tween-20 in PBS. Antibody incubations were performed for 30 min with mouse anti-Cdx2 (Biogenex) at 1:200 and then donkey anti-mouse Alexa Fluor 488 (Molecular Probes) at 1:500. FACS sorting was performed on a FACSAria Cell Sorter 2.0, and data analysed using FlowJo software.

### ChIP analysis of histone modifications

Chromatin immunoprecipitation (ChIP) was performed on native chromatin extracted from 2 × 10^7^ ES or 1 × 10^7^ TS cells using standard protocols (40). Nuclei were purified on a sucrose gradient and chromatin digested with 60 U/ml Micrococcal Nuclease (Affymetrix). Lysates were pre-cleared with Protein G Sepharose beads (GE Healthcare) and incubated with 4 μg of either rabbit anti-H3K9me3 (Abcam ab8898) or rabbit anti-H3K27me3 (Millipore 07–449) at 4°C overnight. Chromatin was immunoprecipitated with Protein G Sepharose beads at 4°C for 4 h. Mock ChIPs were performed in parallel with an isotype-matched control IgG or with beads alone. Eluted DNA from bound and input fractions was subjected to quantitative polymerase chain reaction (PCR) analysis with primer sets specific to genomic promoter regions. Enrichment values were expressed as bound:input ratios and normalized against the corresponding mock values. All ChIPs were performed on at least three biological replicates and compared by T-test. All primers are given in the Supplementary Material.

### ChIP analysis of Parp1 and Parp7

For ChIP analysis of Parp1, both wildtype J1 ES cells and an ES cell clone stably expressing a C-terminally FLAG-tagged Parp1 protein at approximately equal levels to the endogenous protein were used with antibodies against endogenous Parp1 (Santa Cruz Biotechnology sc-74469x) and FLAG (M2, Sigma F1804), respectively. Both approaches yielded highly similar results, except that the anti-FLAG antibody was often more efficient in pull-down. Since the antibody against Parp7 was not of ChIP grade, only anti-FLAG ChIP was performed on ES cell lines stably expressing FLAG-tagged Parp7. Anti-FLAG ChIP on wildtype (vector-only) ES cells and isotype-matched IgG ChIP on Parp1/7-FLAG ES cells were used as controls. Chromatin was cross-linked with 1% formaldehyde, for Parp7 also with 2 mM di(*N*-succinimidyl)glutarate (Sigma), for 10 min, and sonicated to yield ∼200–500 bp fragments. Protein G Sepharose beads were pre-incubated with 5–10 μg of antibody or an isotype-matched control IgG at 4°C for 4 h. Three hundred microgram of chromatin per ChIP was incubated with antibody-bound beads at 4°C overnight. DNA from bound and input fractions was subjected to quantitative PCR analysis, and enrichment values were expressed as percent (%) input. All ChIPs were performed on at least three biological replicates and analysed by two-way ANOVA followed by Holm–Sidak's test for pairwise comparisons.

### RT-qPCR analysis

Total RNA was extracted from cultured cells using Trizol reagent (Invitrogen) and from FACS-sorted cells with RNeasy Micro Kit (QIAgen). cDNA synthesis was performed on 2 μg RNA with H− M-MuLV Reverse Transcriptase (Fermentas). Gene expression was analysed using intron-spanning primer sets and SYBR Green Jump Start Ready Mix (Sigma) on an ABI Prism 7700 or Biorad CFX-96 Real-Time PCR Detection System. Analysis was performed on at least three replicate samples and Ct values normalized against reference genes with the most stable expression across tested samples and compared by T-test or ANOVA, as appropriate.

### DNA methylation analysis

DNA isolated from cultured and FACS-sorted ES cells was processed using the Epitect kit (QIAgen) according to the manufacturer's instructions. Genomic regions were amplified in single or nested PCR reactions using previously described primers for *Elf5* (32) and *Oct4* (41)*;* all primer sequences are given in the Supplementary Material. PCR products were cloned into the pGEM T-Easy Vector (Promega) and sequenced. For analysis by Sequenom Epityper, PCR products were processed with a MassCLEAVE Kit and analysed on a MassARRAY Analyser. Bisulphite data were analysed with the Fisher Exact probability test, and Sequenom data by T-test.

## Results

### Expression of Parp family members in ES and TS cells

**Figure 1.**
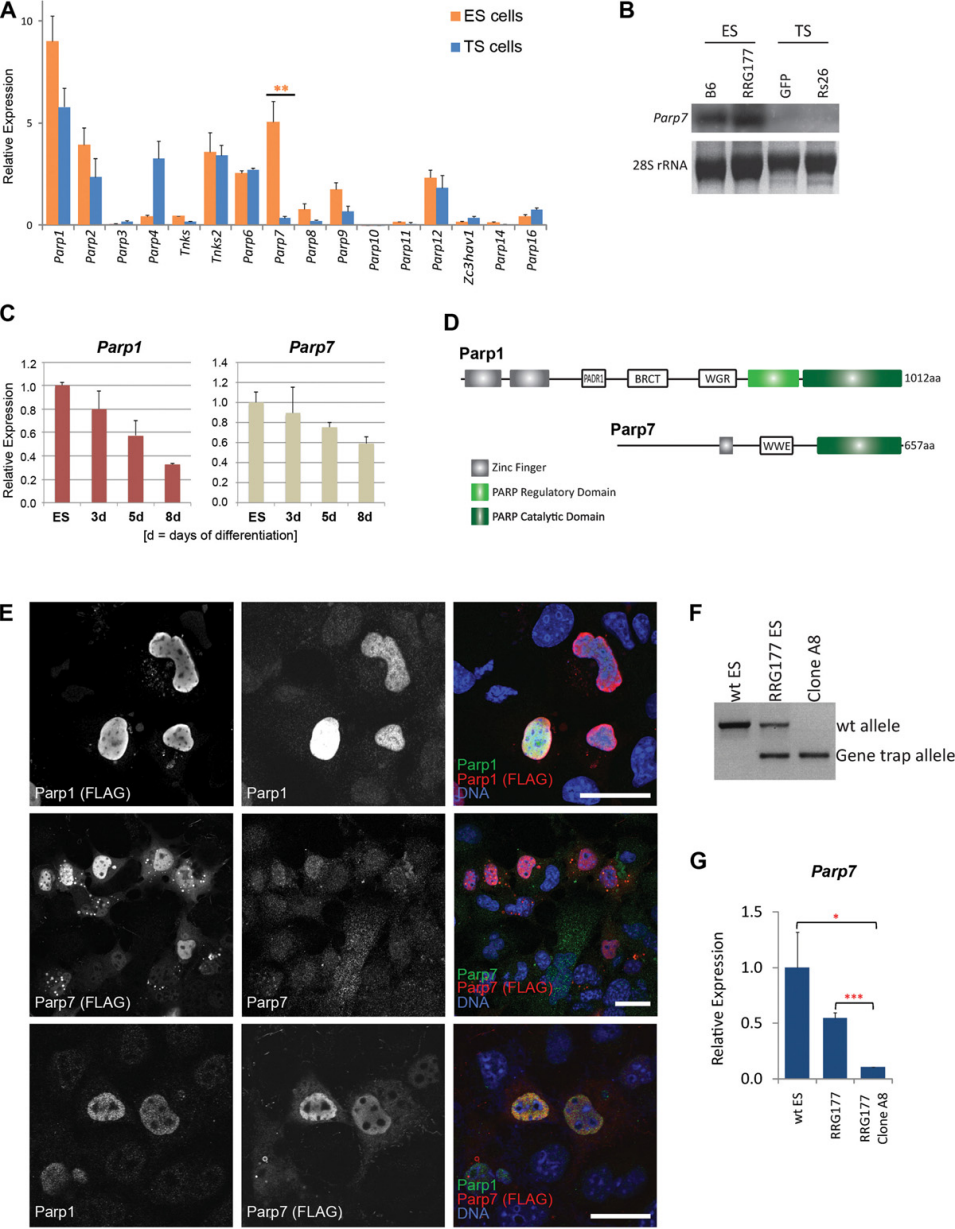
Identification of Parp1 and Parp7 as ES cell-associated genes. (**A**) RT-qPCR analysis of Parp family members in ES and TS cells. Data of 3–6 independent biological replicates are represented as mean + SEM (**P < 0.01). (**B**) Northern blot analysis of *Parp7* expression in two different ES (B6 and RRG177) and TS (Rs26 and GFP) cell lines. (**C**) RT-qPCR analysis of *Parp1* and *Parp7* expression in wildtype (J1 and E14) ES cells over a differentiation time course of 8 days induced by LIF withdrawal and culture on non-adherent plates. (**D**) Schematic representation of the Parp1 and Parp7 protein domain structure. PADR1 = PADR1 domain; BRCT = BRCA1 C-Terminus domain; WGR = WGR domain; WWE = WWE domain. (**E**) Immunolocalization of Parp1 and Parp7, detected by confocal microscopy after staining with antibodies against the C-terminal FLAG-tag as well as against the endogenous proteins, showing the nuclear localisation of Parp1 and Parp7. Note that the antibody against endogenous Parp7 is not particularly efficient. Scale bar: 25 μm. (**F**) Genotyping PCR proving establishment of a homozygously gene-trapped ES cell line at the *Parp7* locus. (**G**) RT-qPCR expression levels of *Parp7* in wildtype (wt), *Parp7*^+/−^ and *Parp7^-^*^/*–*^ ES cells (*P < 0.05, ***P < 0.001).

Previous evidence showed that *Parp1*-deficiency widens the developmental potency of ES cells to include the trophoblast lineage, but that the frequency of transdifferentiation is relatively low (26). To determine whether other Parp's contribute to ES cell maintenance, we first examined the expression of all 16 Parp genes present in the mouse genome (42,43) in ES and TS cells (Figure 1A). RT-qPCR analysis revealed that, overall, *Parp1* was the most abundant of all *Parp*'s in both stem cell types, with higher levels in ES than in TS cells. The most differential expression between the two stem cell types, however, was exhibited by *Parp7* (also known as *Tiparp* or *Artd14* (2)) that was significantly more abundant in ES than in TS cells (Figure 1A and B). Both *Parp1* and *Parp7* were associated with the pluripotent state of ES cells and were down-regulated upon ES cell differentiation (Figure 1C), consistent with their direct regulation by pluripotency genes (44,45). This effect was particularly robust for *Parp1* which decreased to ∼35% of ES cell levels over an 8-day differentiation time course; abundance of *Parp7* transcripts followed the same trajectory, but exhibited an overall greater level of variability depending on precise culture condition. We also assessed expression of the two isoforms of the Parp antagonising enzyme poly-ADP-ribose glycohydrolase (*Parg*), but found no differential expression between ES and TS cells (not shown).

Based on the highly differential expression of *Parp7* between ES and TS cells, we included this factor together with *Parp1* in our further analyses. *Parp7* is a relatively poorly characterized member of the Parp superfamily that was originally cloned as a gene strongly up-regulated upon exposure to halogenated aromatic hydrocarbons (46). Parp7 contains the conserved Parp catalytic domain, a zinc finger motif that may confer DNA binding and a WWE domain that may mediate protein–protein interactions (Figure 1D), and exhibits mono-ADP-ribosylating activity towards itself as well as histones *in vitro* (46,47). Immunostaining experiments demonstrated that both Parp1 and Parp7 are located to the nucleus in a dispersed, largely overlapping pattern (Figure 1E and Supplementary Figure S1A), whilst Parp7 was also detected in a cytoplasmic vesicular fraction in some cells.

### Decreased pluripotency features of Parp1- and Parp7-deficient ES cells

**Figure 2.**
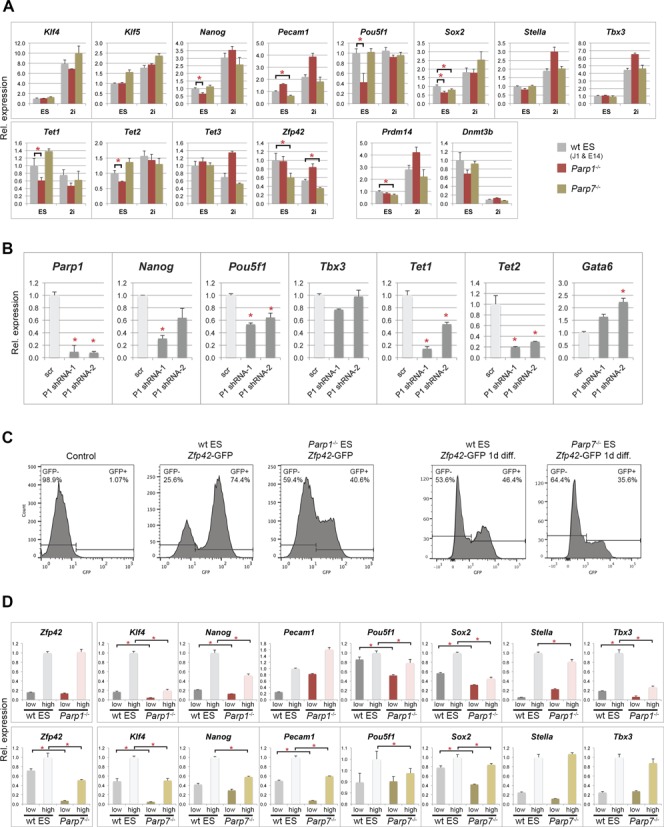
Reduced pluripotency marker expression in *Parp1*^–^^/*–*^ and *Parp7*^–^^/–^ ES cells. (**A**) RT-qPCR analysis of pluripotency markers in wildtype (wt; combined values of J1 and E14 ES cells), *Parp1*^–^^/–^ and *Parp7*^–^^/*–*^ ES cells grown in standard ES cell conditions or in ES cell media containing a MEK and GSK3 inhibitor (‘2i’) (*P < 0.05). *Prdm14* and *Dnmt3b* were used as genes particularly responsive to 2i conditions by being up- and down-regulated, respectively (53). (**B**) Effect of combined deficiency of Parp1 and Parp7, tested in *Parp7*^–/–^ ES cells transfected with two different shRNA constructs against *Parp1* (P1 shRNA-1 and -2) and a scrambled control (scr). *Parp1*, *Pou5f1*, *Tet1* and *Tet2* are significantly down-regulated, and so is *Nanog* with at least one of the constructs. By contrast, the endoderm differentiation marker *Gata6* is up-regulated. (**C**) Flow cytometric analysis of GFP-negative control ES cells, and wt, *Parp1*^–^^/–^ and *Parp7*^–/–^ ES cells carrying a *Zfp42* (= *Rex1*)-GFPd2 knock-in construct that serves as reporter of *Zfp42* expression (38). The proportion of *Zfp42*-GFP^+^ cells is significantly reduced in the absence of *Parp1* or *Parp7*. Note that wt and *Parp7^–^*^/–^ ES cells analysed after one day of LIF withdrawal (‘1d diff.’) were sorted on a different FACS instrument. In each set the gates were adjusted against the appropriate controls. (**D**) RT-qPCR analysis of pluripotency gene expression levels in the separated *Zfp42*-GFP-low and -high cell fractions. Expression levels of most pluripotency markers are reduced, in particular in the *Zfp42*^+^/GFP-high fraction, in both *Parp1*- and *Parp7*-deficient ES cells.

To analyse the functions of *Parp1* and *Parp7*, we obtained gene-trapped *Parp7*^+/–^ ES cells (‘RRG177’), made them homozygously mutant by exposure to high G418 levels (Figure 1F and G) (48) and used them alongside *Parp1*^–/–^ ES cells in all subsequent experiments (25,49). As higher *Parp1* and *Parp7* transcript levels in undifferentiated ES cells indicated a possible role in pluripotency (Figure 1C), we first tested *Parp1*- and *Parp7*-deficient ES cells for the expression of pluripotency markers (50). In line with previous reports (51), we found *Pou5f1* (encoding the Oct4 protein) strongly down-regulated in *Parp1*^−/−^ ES cells to ∼40% of wildtype (wt) levels (Figure 2A). Moreover, we also observed a significant reduction in the expression of *Nanog*, *Sox2*, *Tet1* and *Tet2* to 60–70% of wt levels (Figure 2A). In contrast, *Parp7*-deficiency had a less obvious and more variable impact on pluripotency gene expression in bulk culture; most prominently affected was the expression of *Zfp42* (also known as *Rex1*), *Pecam1*, *Sox2* as well as *Prdm14* (reduced to ∼60–80% of wt levels). When *Parp1* was depleted by shRNA-mediated knockdown in *Parp7*^−/−^ ES cells, the strong down-regulation of *Pou5f1*, *Nanog*, *Tet1* and *Tet2* was again observed (Figure 2B), indicating that Parp1 and Parp7 have additive effects in maintaining pluripotency gene expression. Thus, both *Parp1*- and *Parp7*-deficiency negatively impact on pluripotency gene expression, albeit perhaps with slightly different target gene efficiencies. While the down-regulation of *Zfp42* in *Parp7*^−/−^ ES cells could largely be rescued by ectopic expression of a *Parp7* transgene, the *Parp1*-depletion effect on *Pou5f1* and *Tet1* was not readily reversible in short-term experiments, indicating that more stable, epigenetic changes had occurred at these loci (Supplementary Figure S1B).

To discriminate whether the partial loss of pluripotency features was due to an intrinsic decline in developmental potency or an increased susceptibility to differentiation-promoting signals, we cultured the mutant ES cell lines and their corresponding wt controls in ES media in the presence of a MEK and GSK inhibitor, commonly known as ‘2i’ conditions (52). Expression of *Prdm14* and *Dnmt3b* was up- and down-regulated in these conditions, respectively, as expected (53) (Figure 2A). Notably, pluripotency gene expression levels in *Parp1*-deficient ES cells cultured in 2i reached, or even exceeded, that of wt ES cells, indicating that the decline in pluripotency was largely reversible upon inhibition of pro-differentiation signalling pathways. On the contrary, the reduction of *Zfp42* expression in *Parp7*^–/–^ ES cells staid almost unchanged at 66% of wt cells cultured in parallel, and suggests that ablation of *Parp7* induces a more intrinsic loss of naïve pluripotency (Figure 2A).

Since the observed loss-of-pluripotency phenotype was relatively subtle, we generated knock-in cell lines with a destabilized GFP construct inserted into the *Zfp42*/*Rex1* locus (38) to further substantiate our results. This procedure allowed to accurately quantitate and capture the relative proportion of cells fluctuating between the *Zfp42*-positive and -negative states. When *Zfp42*^+^ cells were FACS-sorted, replated and sorted again after 5 days of culture, it was evident that the proportion of *Zfp42*^+^ cells on the *Parp1*^–/–^ background was significantly lower than in wt ES cells (Figure 2C). Similarly, for *Parp7*^–/–^, more cells were *Zfp42*-negative upon LIF withdrawal for one day, indicative of a more rapid loss of naïve pluripotency features in the absence of Parp7 (Figure 2C). We then collected the *Zfp42*^+^ (GFP-high) and *Zfp42*^−^ (GFP-low) fractions and assessed again the expression of the most prominent pluripotency-associated genes (50) on these separated cell populations. This refined procedure revealed even more clearly that a large cohort of pluripotency genes, including those that were seemingly unchanged on the whole-population level, was significantly down-regulated in the absence of *Parp1* and *Parp7*, in particular in the *Zfp42*^+^ population (Figure 2D). Collectively, these results showed that *Parp1* and *Parp7* contribute to maintaining the naïve state of pluripotency.

**Figure 3.**
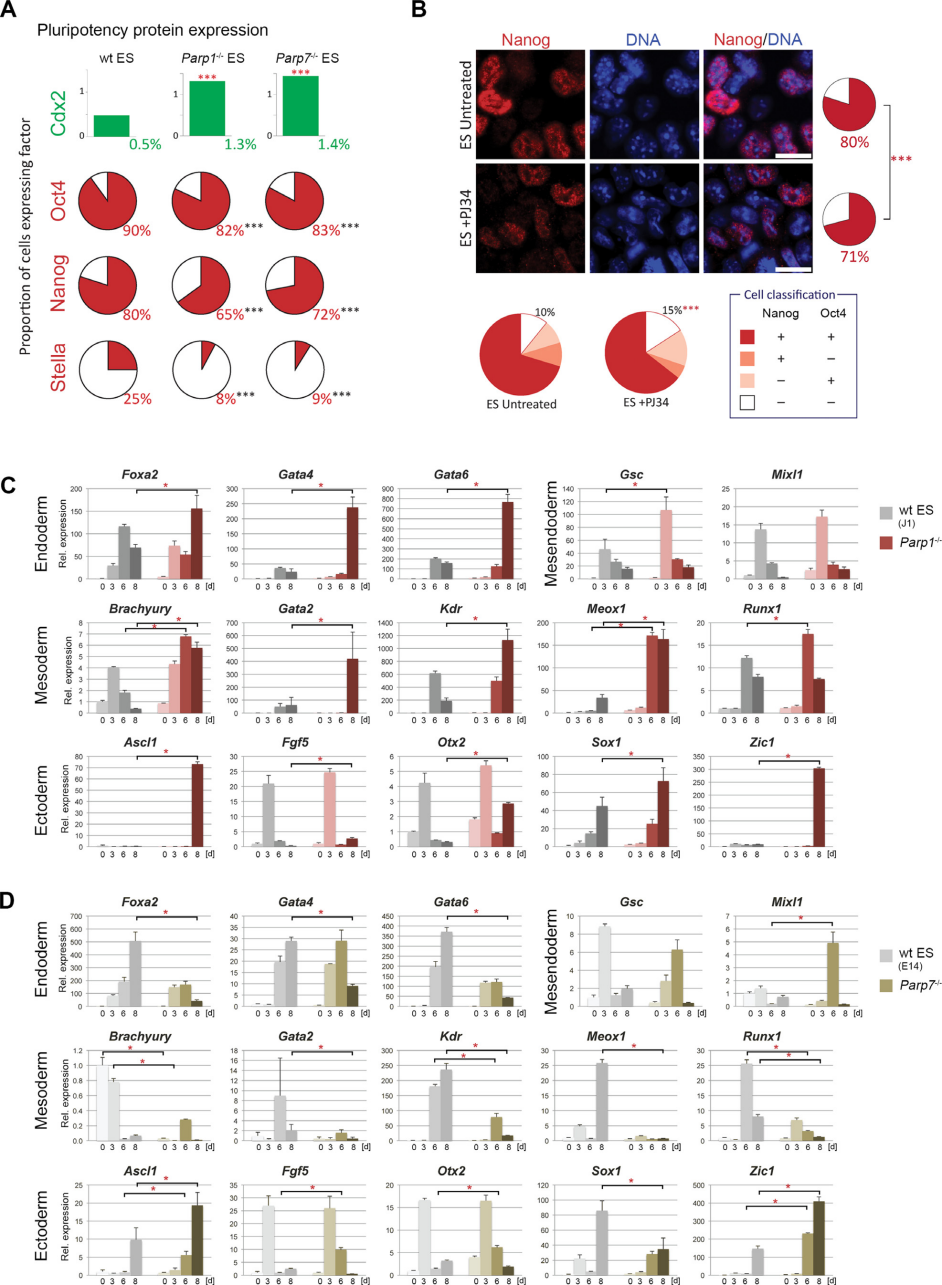
Reduced pluripotency and enhanced differentiation potential of *Parp1^–^*^/–^ and *Parp7*^–/–^ ES cells. (**A**) Quantification of ES cells stained for Cdx2, Oct4, Nanog and Stella, as shown in Supplementary Figure S1. Cells were classified as positive or negative and compared using a Chi-squared test with the Yates correction. Cell numbers analysed were Cdx2: *n* = 8544, 5894 and 5415 for wt (J1 and E14), *Parp1^–^*^/–^ and *Parp7*^–^^/–^, respectively; Oct4: *n* = 2034, 2978 and 715; Nanog: *n* = 1210, 2108 and 1032; Stella: *n* = 1385, 1555 and 1278; *P* < 0.001 in all cases. (**B**) Immunofluorescence staining and quantification for Nanog and Oct4 in wt ES cells cultured for 4 days in ES conditions in either the presence or the absence of the poly-ADP-ribose polymerase inhibitor PJ34. A very similar reduction in pluripotency factor expression is observed. Cell numbers were: Nanog: *n* = 2476 and 2477; *P* < 0.001; Oct4: *n* = 2133 and 1992; *P* = 0.42; Nanog/Oct4 double staining: *n* = 1718 and 1045; *P* < 0.001. Scale bars: 20 μm. (**C**) and (**D**) RT-qPCR analysis of marker gene expression levels in embryoid bodies generated by hanging drop culture. Note the skewed differentiation trajectories in both *Parp1^–^*^/–^ and *Parp7*^–^^/–^ ES cells compared to their background-matched wt control ES cell lines. *Parp1*-deficient embryoid bodies exhibited a greater variability in size and displayed an overall enhanced speed and/or extent of differentiation towards all embryonic lineages. *Parp7* deletion abrogated differentiation towards mesoderm and definitive endoderm (*Foxa2*, *Gata4*, *Gata6*), but overall enhanced ectoderm differentiation.

To identify whether this difference on the mRNA level was reflected on the protein level, we performed a series of immunostainings for Oct4 (encoded by the *Pou5f1* gene), Nanog, Stella (also known as Pgc7 or Dppa3) as well as Cdx2, a transcription factor associated with early trophoblast differentiation in pre- and peri-implantation embryos. A large number of randomly selected cells were analysed and scored for expression (Figure 3A and Supplementary Figure S1C–E). Of note, the proportion of Stella-expressing cells in our wt ES cells correlated well with previous reports (54). In *Parp1*- and *Parp7*-deficient ES cells, however, the proportion of Oct4-, Nanog- and Stella-positive cells was lower, while at the same time the number of Cdx2-positive cells was increased (Figure 3A and Supplementary Figure S1C). These data confirmed that in the absence of *Parp1* and *Parp7*, ES cells exhibit a global decline in pluripotency hallmarks. Co-immunofluorescence stainings for Cdx2 and Oct4, Nanog or Stella further revealed that on the individual cell level, Cdx2 expression was not correlated with Oct4 down-regulation (Supplementary Figure S1D and E). However, Cdx2 expression was strictly confined to Nanog- and Stella-negative cells, showing that these pluripotency factors are critical sensors of the naïve state of ES cell potency (Supplementary Figure S1E). This correlation was evident irrespective of ES cell genotype, even though the Cdx2^+^ fraction was significantly smaller in wt ES cells than in *Parp1*^−/−^ and *Parp7*^−/−^ ES cells (Figure 3A).

To discriminate between a role of the Parp proteins versus their ADP-ribosylating activity, we compared the effects of genetic *Parp1*- or *Parp7*-deficiency to the chemical inhibition of PARylation by treating wt ES cells with the broad-spectrum Parp inhibitor PJ34 for 4 days. Parp inhibition caused a similar decrease in Nanog and Oct4-positive cells as observed in *Parp1*- and *Parp7*-mutant ES cells (Figure 3B), indicating that much of the observed loss in pluripotent capacity is mediated through the enzymatic activity of these proteins.

### Increased propensity to differentiate in Parp1- and Parp7-deficient ES cells

Next we assessed the differentiation dynamics of *Parp1*- and *Parp7*-deficient ES cells during embryoid body formation (by hanging drop culture; Figure 3C and D) and upon exposure to retinoic acid (Supplementary Figure S2). In line with the reduced expression levels of a cohort of pluripotency-associated genes, *Parp1* null ES cells differentiated more rapidly than their wt counterparts into derivatives of all three germ layers in embryoid bodies, as is evident from the earlier and/or higher induction levels of a number of lineage markers (Figure 3C). In contrast, *Parp7*^–/–^ ES cells exhibited a specific differentiation bias: formation of definitive endoderm and mesoderm derivatives occurred very poorly in embryoid bodies (even though *Parp7*^–/–^ ES cells were principally capable of differentiating into these lineages as revealed by retinoic acid treatment (Supplementary Figure S2)), while differentiation into the ectoderm lineage was accelerated (Figure 3D). Particularly notable was the strong induction of *Ascl1* and *Zic1* in both *Parp1*^-/-^ and *Parp7*^–/–^ embryoid bodies, both genes indicative of neuronal differentiation (55).

**Figure 4.**
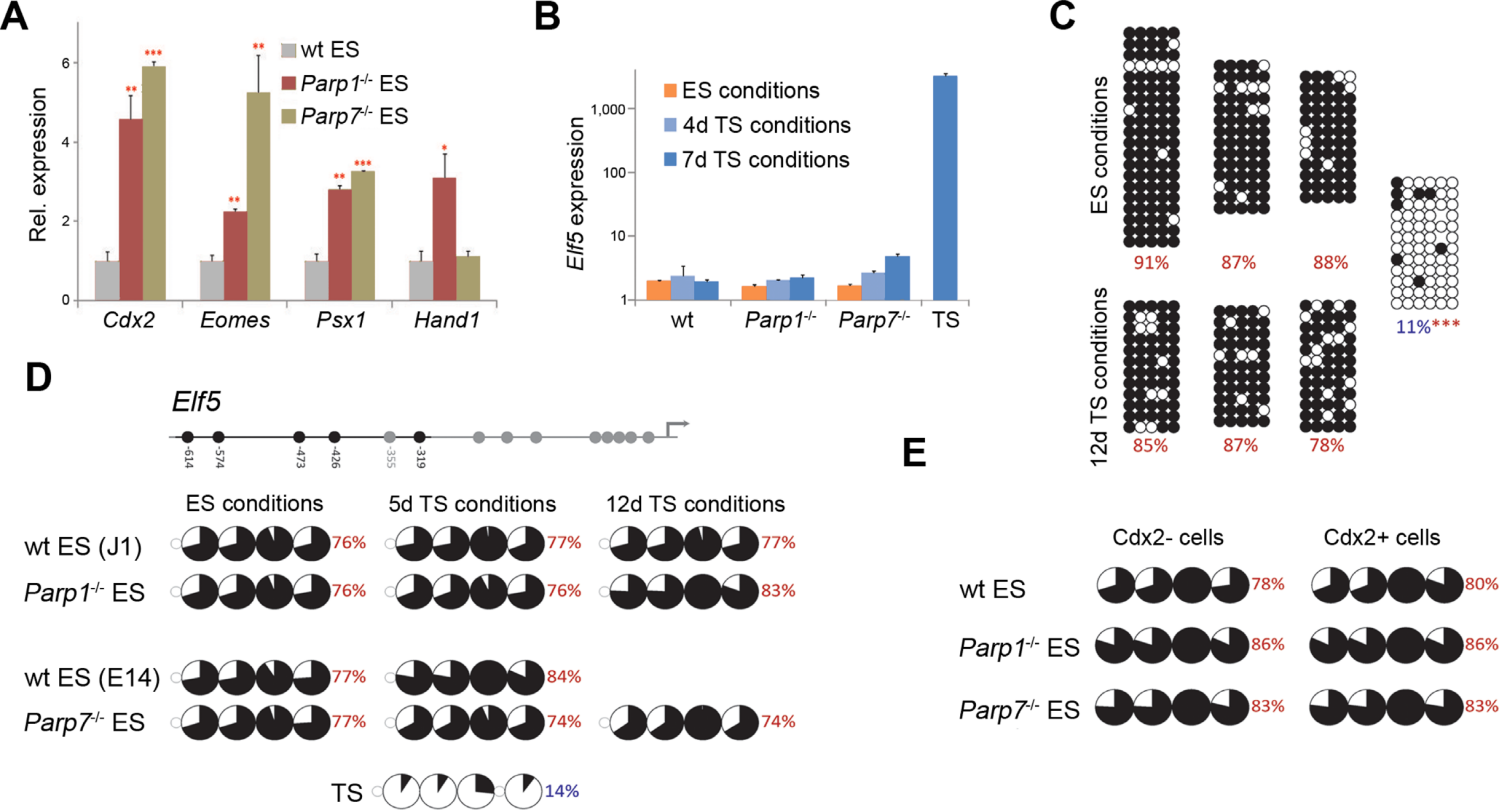
Acquisition of a limited set of TS cell features in the absence of Parp1 and Parp7. (**A**) RT-qPCR analysis of trophoblast marker gene expression in wildtype (wt), *Parp1^-^*^/*-*^ and *Parp7^-^*^/*-*^ ES cells cultured in TS cell medium for four days (*P < 0.05, **P < 0.01, ***P < 0.001). (**B**) RT-qPCR analysis of *Elf5* expression in wildtype (wt; combined values of J1 and E14 ES cells), *Parp1^-^*^/*-*^ and *Parp7^-^*^/*-*^ ES cells cultured in ES cell conditions or in TS cell conditions for 4 or 7 days shows no induction of this trophoblast lineage gatekeeper gene. Expression levels in TS cells are provided for comparison. (**C**) Bisulphite sequencing analysis of the *Elf5* gene promoter. Data were compared using the Chi-squared test (***P < 0.001). (**D**) Sequenom Epityper analysis of *Elf5* promoter methylation. Pie charts represent average methylation levels at each CpG dinucleotide. The CpG units captured are indicated in the gene graph. (**E**) Sequenom Epityper analysis of *Elf5* DNA methylation in wt, *Parp1^-^*^/*-*^ and *Parp7^-^*^/*-*^ ES cells cultured in ES cell conditions, stained for Cdx2 and FACS-sorted into Cdx2^+^ and Cdx2^−^ cell populations.

Based on our previous findings that *Parp1*-deficient ES cells can differentiate into trophoblast derivatives (26) and that *Parp1*^–/-^ and *Parp7*^-/-^ ES cells contain greater numbers of Cdx2-positive cells (Figure 3A), we determined their differentiation capacities towards the trophoblast lineage in more detail. When assessing their behaviour upon shift from ES to TS cell culture conditions (consisting of embryonic feeder cell-conditioned medium and bFGF, but no LIF), we indeed detected a significant up-regulation of the TS cell factors *Cdx2* and *Eomes*, together with markers of differentiated trophoblast cell types such as *Psx1* (Figure 4A). Up-regulation of *Hand1* only in *Parp1*^-/-^ ES cells suggested subtle differences in the precise timing of differentiation towards giant cell-like cells (56). These data extend previous reports of the occurrence of differentiated trophoblast giant cells from *Parp1*^−/−^ ES cells; they indicate an up-regulation of genes commonly associated even with early trophoblast commitment and TS cell proliferation. *Parp7*-deficiency seemingly causes a similar phenotype, a finding corroborated by the higher fraction of cells with flattened, epithelial-like morphology in these cultures (Supplementary Figure S3A). In line with the reduction in pluripotency gene expression, PJ34-treatment of wt ES cells also caused an activation of *Cdx2* to the same level as in *Parp1-* and *Parp7*-deficient ES cells (Supplementary Figure S3B).

### Elf5 methylation is not affected by Parp1- and Parp7-deficiency

The flattened appearance and expression of trophoblast-associated genes in *Parp1- and Parp7*-deficient ES cells raised the question whether these cells harboured true TS cell capacity. Perhaps the most informative marker to make this stem cell-type distinction is the transcription factor *Elf5*, a gene we identified previously as an epigenetically regulated lineage ‘gatekeeper’ crucial for trophoblast cell fate commitment (32). *Elf5* is methylated and repressed in ES cells, but hypomethylated and highly expressed in TS cells. Conversion of ES into genuine TS cells thus requires demethylation and activation of the *Elf5* locus. In contrast to the situation in hypomethylated ES cell models, however, *Elf5* was not up-regulated and correspondingly, remained fully methylated in *Parp1*^-/-^ and *Parp7*^-/-^ ES cells, indistinguishable from wt ES cells (Figure 4B–D). This epigenetic status was evident also when the Cdx2-positive cell fraction was assessed separately, largely ruling out the possibility of a small cell population hypomethylated at *Elf5* (Figure 4E). Given that *Elf5* is instrumental for TS cell derivation and maintenance (57), this lack of epigenetic reprogramming and transcriptional activation of *Elf5* likely explains why no outgrowing, proliferative TS-like colonies could be isolated from *Parp1*^-/-^ and *Parp7*^-/-^ ES cells even when they were cultured in TS cell conditions over prolonged periods of time.

### Parp1 and Parp7 maintain an active chromatin configuration at pluripotency genes

**Figure 5.**
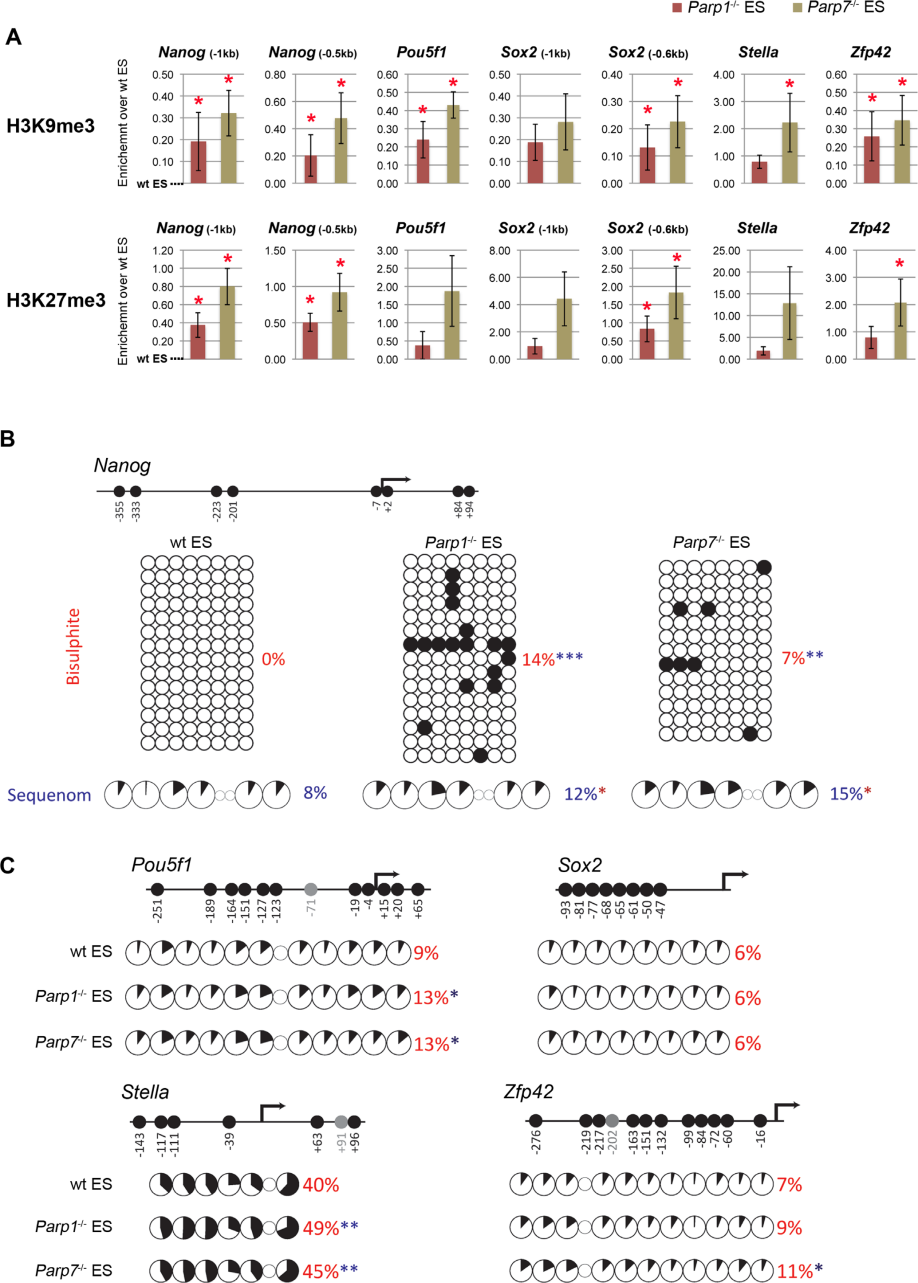
Accumulation of epigenetic repressive marks at pluripotency factor loci in *Parp1^-^*^/*-*^ and *Parp7^-^*^/*-*^ ES cells. (**A**) ChIP against histone modifications H3K9me3 and H3K27me3 in wildtype (wt), *Parp1^-^*^/*-*^ and *Parp7^-^*^/*-*^ ES cells cultured in ES cell conditions. Fold enrichment values were calculated from the ratio of bound to input DNA, and corresponding values from control IgG ChIPs were subtracted. Data are displayed with values in wt ES cells set to 0. (**B**) DNA methylation at the *Nanog* promoter by bisulphite sequencing and Sequenom Epityper analysis. The analysed region is depicted in the schematic of the locus. (**C**) Sequenom Epityper analysis of DNA methylation at the *Pou5f1*, *Sox2*, *Stella* and *Zfp42* promoters. Graphic representations of the CpG distribution and those CpG dinucleotides analysed are indicated for each gene (*P < 0.05, **P < 0.01, ***P < 0.001).

To explain the reduction of ground state pluripotency and the concomitant increase in the propensity to differentiate in *Parp1*- and *Parp7*-deficient ES cells, we examined the epigenetic state of pluripotency gene loci. When assessed by ChIP assays, we detected a significant increase in the repressive histone modifications histone H3 lysine 9 and lysine 27 trimethylation (H3K9me3 and H3K27me3) at many of these loci (Figure 5A). We also detected a subtle yet consistent and significant increase in DNA methylation at the *Nanog*, *Pou5f1*, *Stella* and *Zfp42* promoters (Figure 5B and C). These increases were detected by bisulphite sequencing as well as by Sequenom mass array that measures average DNA methylation levels across all bisulphite-treated DNA fragments of the entire cell population (Supplementary Figure S4). The clonal information contained in the bisulphite sequences indicated a stochastic acquisition of DNA methylation at individual CpG dinucleotides in *Parp1*^-/-^ and *Parp7*^-/-^ cells, suggesting a progressive repression of these loci by a gradual increase in repressive histone modifications as well as DNA methylation.

### Parp1 and Parp7 safeguard the metastable state of pluripotency

**Figure 6.**
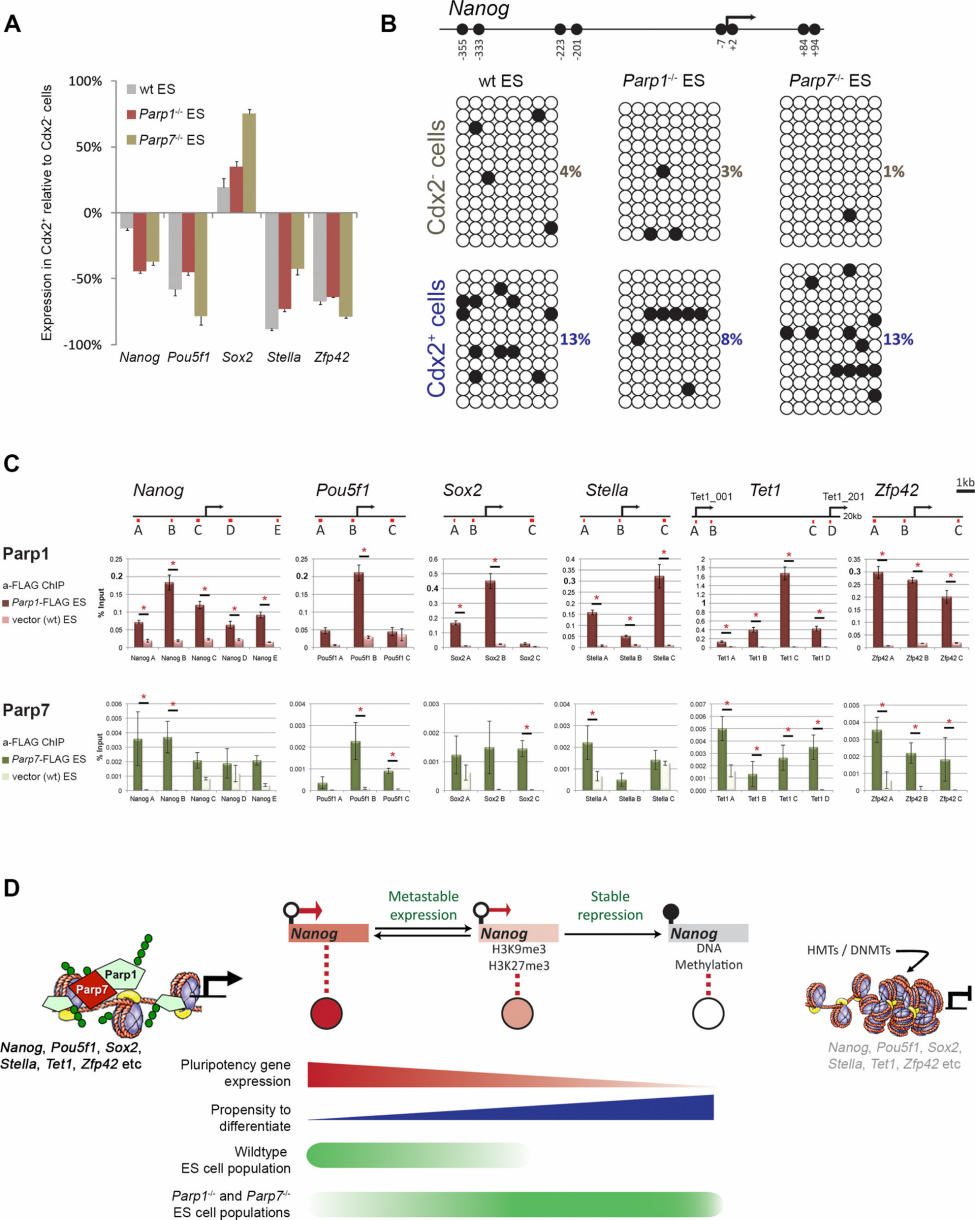
Parp1 and Parp7 contribute to maintaining the metastable state of pluripotency. (**A**) RT-qPCR analysis of pluripotency gene expression in wildtype (wt), *Parp1^-^*^/*-*^ and *Parp7^-^*^/*-*^ ES cells cultured in ES cell conditions and FACS-sorted into Cdx2-negative (Cdx2^−^) and -positive (Cdx2^+^) populations. Expression changes are calculated as the percentage difference between Cdx2^+^ and Cdx2^−^ cell populations. (**B**) Bisulphite sequencing profiles of the *Nanog* locus (as depicted in Figure 5B) in the Cdx2^−^ and Cdx2^+^ fractions of wt, *Parp1^-^*^/*-*^ and *Parp7^-^*^/*-*^ ES cells. (**C**) ChIP analysis of pluripotency locus occupancy by Parp1 and Parp7 using ES cells stably expressing C-terminally FLAG-tagged Parp1 and Parp7 constructs at approximately equal levels to endogenous proteins (Supplementary Figure S5). Chromatin was cross-linked with formaldehyde (for Parp1) and additionally with 2 mM di(*N*-succinimidyl)glutarate (for Parp7). For Parp1, ChIP was also performed against the endogenous protein on wt ES cells (Supplementary Figure S6). As controls, ChIPs against FLAG on wt ES cells, and with isotype-matched IgG on Parp1-FLAG/Parp7-FLAG cell lines were performed (Supplementary Figure S6). Schematic diagrams show the genomic locations of primer sets (red lines) used. All primers are given in the Supplementary Material. (*P < 0.05) (**D**) Model of Parp1 and Parp7 function in maintaining ES cell pluripotency. ES cells are normally in a dynamic equilibrium between higher and lower states of potency that are characterized by distinct histone modifications at key loci such as *Nanog*, *Stella*, *Pecam1* and *Zfp42*. Parp1 and Parp7 preserve this dynamic equilibrium; in their absence, ES cells are more likely to acquire epigenetic repressive marks at pluripotency gene loci, including DNA methylation, which commits them towards differentiation. Green circles depict ADP-ribose moieties, which may be found on Parp1 and Parp7 themselves (auto-PARylation) or deposited on the immediate histone environment. Since PAR moieties introduce negative charges, a scenario of gentle electrostatic repulsion to relax the DNA fibre can be imagined as a mechanism to preserve an open chromatin structure or to repel binding of repressive factors. HMTs = histone methyltransferases, DNMTs = DNA methyltransferases.

The fluctuation in expression of pluripotency genes such as *Nanog, Stella* and *Zfp42* in normal ES cell populations has led to the notion that ES cells are in a metastable state characterized by distinct yet reversible changes between active and repressive histone modifications at these loci (54). By contrast, DNA methylation of these genes demarcates an irreversible exit from the pluripotent state. To distinguish between these possibilities in *Parp1-* and *Parp7*-deficient ES cells, we analysed DNA methylation levels at key pluripotency gene promoters specifically in the Cdx2-positive (and hence Nanog/Stella/Zfp42-negative) cell population. This experimental set-up allowed us to assess ES cells at the margin of the metastable state irrespective of their relative frequency in all three genotypes (wt, *Parp1*^-/-^ and *Parp7*^-/-^), even though the absolute number of Cdx2^+^ cells in wt ES cells is very small. The Cdx2^+^ populations were highly enriched for cells with lower pluripotency gene expression of *Nanog*, *Pou5f1*, *Stella* and *Zfp42*, as expected (Figure 6A). Conversely, they exhibited higher DNA methylation levels at these loci (Figure 6B, Supplementary Figure S4), demonstrating that the overall methylation changes detected were largely confined to the Cdx2^+^ cell population. Collectively, these data show that *Parp1*^-/-^ and *Parp7*^-/-^ ES cells exhibit a decreased ability to maintain the metastable state and contain a significant proportion of cells committed to differentiate as they acquire both reversible and stable epigenetic repressive marks at key pluripotency genes.

To explain how pluripotency is maintained by Parp1 and Parp7, we considered whether they directly bind to pluripotency gene loci and thereby protect them from epigenetic repression. We therefore tested the genomic occupancy of Parp1 and Parp7 by ChIP using primer pairs broadly surrounding the transcriptional start sites of key pluripotency loci. For this purpose, stable ES cell lines were generated expressing C-terminally FLAG-tagged Parp1 and Parp7 constructs at ∼1–2x levels of the endogenous protein (Supplementary Figure S5). For Parp1, ChIP was performed against the endogenous protein in wt ES cells as well as against the FLAG-tagged version in stable cell lines. Both approaches yielded comparable results, as expected (58,59), except that the anti-FLAG ChIP was overall more efficient (Supplementary Figure S6A and B). For Parp7, poor antibody quality against the endogenous protein allowed us to only perform anti-FLAG ChIP on stably transfected cell lines (using IgG, and anti-FLAG ChIP in wt cells, as controls (Supplementary Figure S6C)). Overall, we found that both Parp1 and Parp7 broadly occupy pluripotency loci. Specifically high enrichment was observed for Parp1 around the *Nanog*, *Pou5f1*, *Sox2*, *Tet1* and *Zfp42* transcriptional start sites. In line with its previously demonstrated DNA binding capacity (47), Parp7 was also found associated with pluripotency gene loci albeit at relatively low enrichment compared to Parp1 (Figure 6C; Supplementary Figure S6D). These results showed that both Parp's occupy pluripotency gene loci and thereby protect them from epigenetic repression to preserve the developmental plasticity of ES cells.

## DISCUSSION

Recent insights have highlighted the importance of the poly-ADP-ribose polymerase Parp1 in gene specific as well as higher-order chromatin organisation and in epigenetic reprogramming (60,61). In the context of somatic cell reprogramming, Parp1 has been shown to have a beneficial effect by promoting the accessibility of pluripotency factors to their DNA target sites (20). This may be conferred by Parp1's ability to substitute for the linker histone H1 at active loci to maintain an open chromatin configuration (9). Analysis of the *Dnmt1* promoter has further suggested that Parp1 can protect this locus from epigenetic repression by DNA methylation (11). In the present study, we show that this function of Parp1 is specifically important to maintain the pluripotent nature of ES cells by ensuring the transcriptional activity of key pluripotency genes such as *Nanog*, *Pou5f1*, *Sox2*, *Tet1* and *Tet2*. In addition, we find a similar yet not identical role for another Parp family member, Parp7.

Parp7 is a relatively poorly characterized Parp, which was first identified as highly induced upon exposure to 2,3,7,8-tetrachlorodibenzo-p-dioxin (TCDD). Unlike the poly-ADP-ribosyltransferase Parp1, Parp7 has recently been found to exhibit mono-ADP-ribosylation activity both against itself and against histones (46,47,62). Corroborating previous reports, we find Parp7 to be mostly localized to the nucleus, in a dispersed pattern largely overlapping with Parp1, and to exhibit DNA binding capacity (47). Similar to Parp1, Parp7 is broadly associated with active loci, albeit at relatively low enrichment, which may reflect the overall less prevalent effects on pluripotency features in *Parp7* null cells compared to *Parp1*-deficiency. In general, the sites co-occupied by Parp1 and Parp7 often harbour key regulatory features and may form transcription factor hubs; this is exemplified by the Parp1- and Parp7-enriched regions surrounding the *Nanog* and *Pou5f1* transcription start sites to which Esrrb, Tcfcp2l1, Klf4, Sox2, Oct4 and Nanog binding sites have been mapped.

Despite this broad occupancy of pluripotency loci, our comprehensive assessment of the *Parp1*- and *Parp7*-mutant ES cell phenotypes detected subtle differences in the precise subsets within the pluripotency gene network that are most affected. As such, Parp7 appears to have a more prominent impact on *Zfp42*, *Sox2*, *Pecam1* and *Prdm14* transcript levels, whereas *Parp1*-deficiency led to a strong decrease of *Pou5f1*, *Sox2, Tet1, Nanog* and *Tet2* in bulk culture. Since the pluripotency network is self-regulatory and mutually enhancing, however, these slightly diverging primary effects will lead to similar outcomes, most notably the decline in naïve pluripotency and a lower capacity to maintain the heterogeneity in gene expression characteristic of the ES cell state. Indeed, such widespread impact on pluripotency gene expression was evident when ES cells were separated into *Zfp42*^+^ and *Zfp42*^−^ populations or assessed on the single-cell level population-wide by immunostaining.

The only partial phenocopy of *Parp1*^-/-^ and *Parp7*^-/-^ ES cells can also be seen in the embryoid differentiation experiment in which the behaviour of the two mutant ES cell lines diverges. In line with the broad DNA binding profile and widespread effect on pluripotency gene expression, *Parp1*-deficient ES cells show a generally increased propensity to differentiate into derivatives of all three germ layers. By contrast, ES cells null for *Parp7* exhibit a differentiation bias, with reduced definitive endoderm and mesoderm marker expression but an enhanced differentiation towards (neuro-)ectoderm. Of note is the specifically high induction of the neural differentiation markers *Ascl1* and *Zic1* in both *Parp1*^-/-^ and *Parp7*^-/-^ ES cells. Intriguingly, Parp1 has been previously linked to the neuronal differentiation pathway at the *Ascl1* locus, albeit in a seemingly opposing role where Parp1's catalytic activation was necessary to induce the dissociation of co-repressors and thereby to activate gene expression (63). Our data suggest that Parp1 (and Parp7) are required to repress this pathway to maintain ES cells in an undifferentiated state. It is likely that, similar to the seemingly antagonistic effects of Parp1 on chromatin organisation, this dual function may be regulated by the precise levels of Parp activation (11). Overall, our results demonstrate an important role for both Parp1 and Parp7 in maintaining the developmental plasticity of ES cells by ensuring an open chromatin configuration and high expression level of key pluripotency genes.

A defining hallmark of the ES cell state is its intrinsic transcriptional heterogeneity and metastability, which has been suggested to underlie the differentiation potential into a wide array of different cell types (54). Thus, ES cells constantly fluctuate between states of higher and lower potency as identified by the heterogeneous expression of genes such as *Stella*, *Nanog*, *Pecam1* and *Zfp42* (54,64–66). Cells in which these genes are not expressed are epigenetically distinct as they lack hallmarks of active chromatin at these loci. Yet the expressing and non-expressing cell populations remain inter-convertible unless the epigenetic repression involves DNA methylation, which demarcates the terminal exit from pluripotency. *Parp1*^-/-^ and *Parp7*^-/-^ ES cells accumulate epigenetic repressive marks including DNA methylation at these loci in a stochastic manner; thus, while the mutant ES cell populations on the whole remain pluripotent, they exhibit an overall decrease in the naïve state of pluripotency and, conversely, an increased propensity to differentiate (Figure 6D).

The function of Parp1 and Parp7 in maintaining the transcriptional heterogeneity of ES cells shares some notable similarities with the nucleosome remodelling and deacetylation (NuRD) complex (50). Like Parp1 and Parp7, the NuRD complex has also been shown to modulate the transcriptional heterogeneity and dynamic range of a set of pluripotency genes. However, contrary to the situation in *Parp1* and *Parp7* null ES cells, pluripotency genes become hyper-activated and differentiation is perturbed in the absence of the core NuRD component *Mbd3*. Thus, NuRD and Parp's may co-operate to regulate the expression of pluripotency genes by exerting opposite effects that fine-tune their precise levels. This functional connection is particularly interesting as NuRD and Parp's/PAR are known to physically interact (67,68), and therefore may counterbalance each other to maintain the heterogeneity and metastability characteristic of ground state pluripotency.

These data establish an important role for Parp1 and Parp7 in fine-tuning the core pluripotency network of ES cells (21). Indeed both genes have multiple Oct4, Sox2 and Nanog binding sites (44,45,69,70), and—as we demonstrate here—in turn bind to, and maintain, the activity of these pluripotency genes. Our data are further corroborated by recent reports that showed Parp1 binding to the exon1/intron1 of *Nanog*, a capacity linked to its supportive role in iPS cell reprogramming (20). This role adds another regulatory level to Parp1's involvement in ES cell pluripotency, in addition to its direct PARylation-linked function on the Sox2 protein that may alter Sox2's transcriptional activity. Parp1- and Parp7-mediated protection from epigenetic repression may simply be the result of steric hindrance denying histone and DNA methyltransferases access to chromatin (Figure 6D). Another possibility arises from recent insights that active promoters may require a continuous turnover of stochastically accumulating DNA methylation marks (71,72). DNA demethylation involves the conversion of 5-methylcytosine to 5-hydroxymethylcytosine, a reaction catalysed by the Tet enzymes or by Aid-mediated deamination and subsequent DNA repair (73). Strikingly, knockdown of *Tet1* in ES cells causes a remarkably similar *de novo* methylation pattern of the *Nanog* promoter as we observed in *Parp1*^-/-^ and *Parp7*^-/-^ ES cells (74). Parp1 has recently been proposed to be an important down-stream mediator of the base excision repair processes following active demethylation in ES cells, as well as in the germ line and in the zygote (60,75,76). Thus, it is tempting to speculate that this same mechanism is involved in the continuous turnover of stochastic methylation events in ES cells to maintain ground state pluripotency.

Taken together, we have identified an important function of Parp1 and Parp7 in contributing to early cell fate restriction and in fine-tuning the labile, metastable state inherent to pluripotency. Our insights provide a mechanistic link between these post-translational modifiers and the epigenetic machinery in stem cell plasticity and in the control of differentiation.

## SUPPLEMENTARY DATA

Supplementary Data are available at NAR Online.

SUPPLEMENTARY DATA
