# Supplementary material for: ADP-ribosyltransferases Parp1 and Parp7 safeguard pluripotency of ES cells
Source: Nucleic Acids Res. 2014 Jul 17;42(14):8914–27. doi: 10.1093/nar/gku591 (PMC4132717; doi:10.1093/nar/gku591)
Supplement: SUPPLEMENTARY DATA [file supp_42_14_8914__index.html]

ADP-ribosyltransferases Parp1 and Parp7 safeguard pluripotency of ES cells — SUPPLEMENTARY DATA 

# ADP-ribosyltransferases Parp1 and Parp7 safeguard pluripotency of ES cells

## SUPPLEMENTARY DATA

**Files in this Data Supplement:**

- SUPPLEMENTARY DATA
