# Supplementary material for: ADP-ribosyltransferases Parp1 and Parp7 safeguard pluripotency of ES cells
Source: Nucleic Acids Res. 2014 Jul 17;42(14):8914–27. doi: 10.1093/nar/gku591 (PMC4132717; doi:10.1093/nar/gku591)
Supplement: SUPPLEMENTARY DATA [file supp_gku591_nar-01282-v-2014-File008.pdf]

Roper et al.,

ADP-ribosyltransferases Parp1 and Parp7 safeguard pluripotency of ES cells

Supplementary Material

Supplementary Figure S1

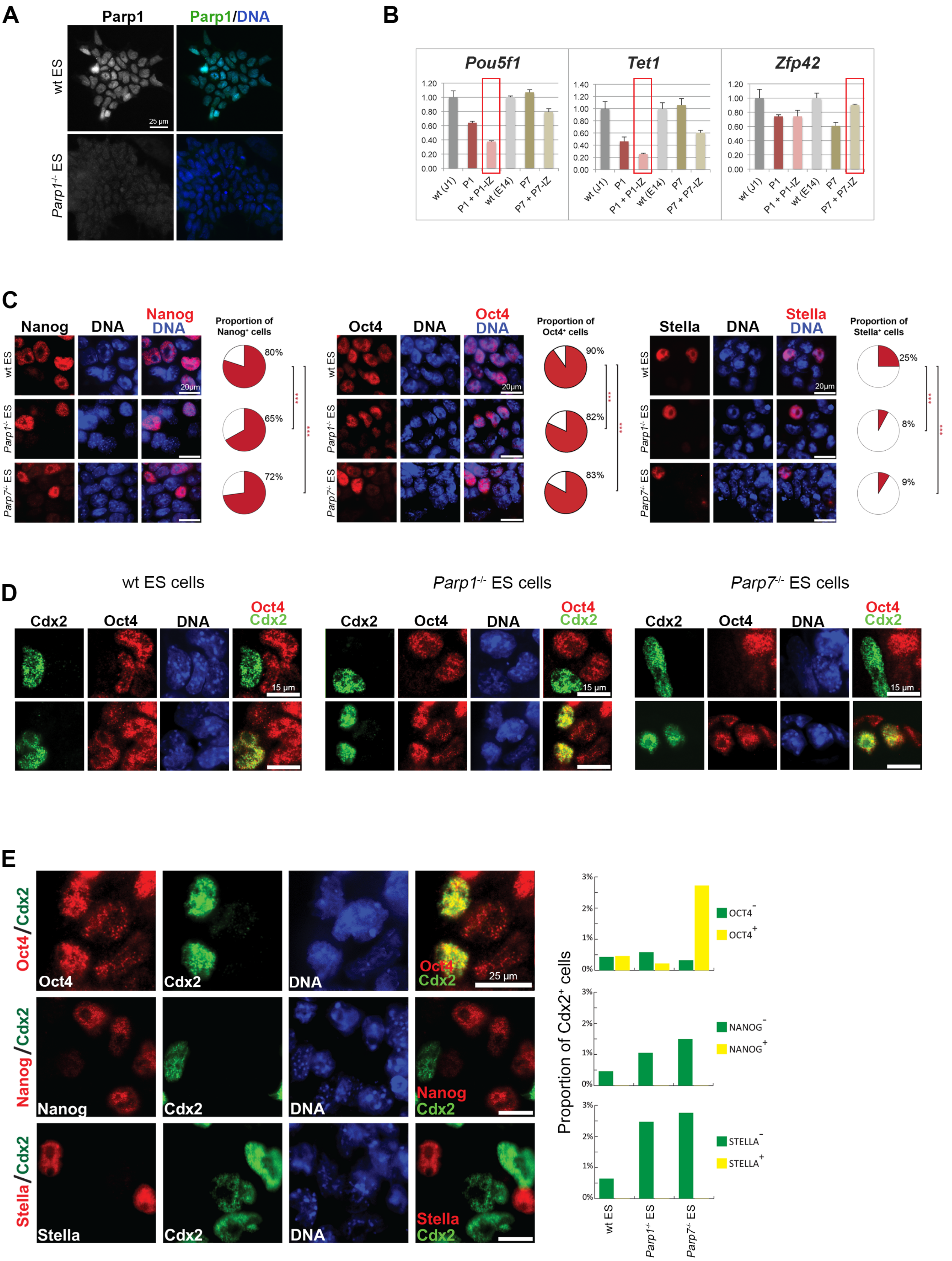

Supplementary Figure S1. Pluripotency gene expression characteristics associated with loss of Parp1 and Parp7. (**A**) Parp1 immunostaining (clone E-8, Santa Cruz Biotechnology sc-74469x) on wildtype (wt) and *Parp1*$^{-/-}$ ES cells demonstrates nuclear localization of Parp1, as expected. (**B**) Rescue experiments reintroducing a *Parp1* ("P1-IZ") and *Parp7* ("P7-IZ") transgene into *Parp1*$^{-/-}$ ("P1") and *Parp7*$^{-/-}$ ("P7") ES cells, respectively. RNA was collected and RT-qPCRs performed after 4 days of antibiotic selection. Samples marked with the red rectangle highlight the lack of rescue of *Pou5f1* and *Tet1* expression in *Parp1*$^{-/-}$ ES cells, while *Zfp42* expression in *Parp7*$^{-/-}$ ES cells reached close to wildtype levels upon reintroduction of Parp7. (**C**) Immunofluorescence staining and cell counts of positive cells in wildtype, *Parp1*$^{-/-}$ and *Parp7*$^{-/-}$ ES cells. Cells were classified as positive or negative for the respective factor and data compared using a Chi-squared test with the Yates correction. Number of analysed cells were; Oct4: n=2,034, 2,978 and 715; Nanog: n=1,210, 2,108 and 1,032; Stella: n=1,385, 1,555 and 1,278; $p<0.001$ in all cases. (**D**) Examples of double immunofluorescence stainings for Cdx2 and Oct4 in wildtype (wt), *Parp1*- and *Parp7*-deficient ES cells. Quantifications shown as part of (D). (**E**) Double immunofluorescence staining for Cdx2 and Oct4, Nanog or Stella, shown on the example of *Parp1*$^{-/-}$ ES cells. Graphs indicate the proportion of cells staining positive for Cdx2 within the Oct4, Nanog and Stella-positive and -negative cell populations. Cdx2 expression was confined exclusively to cells negative for Nanog and Stella, but did not correlate with lack of Oct4.

Supplementary Figure S2

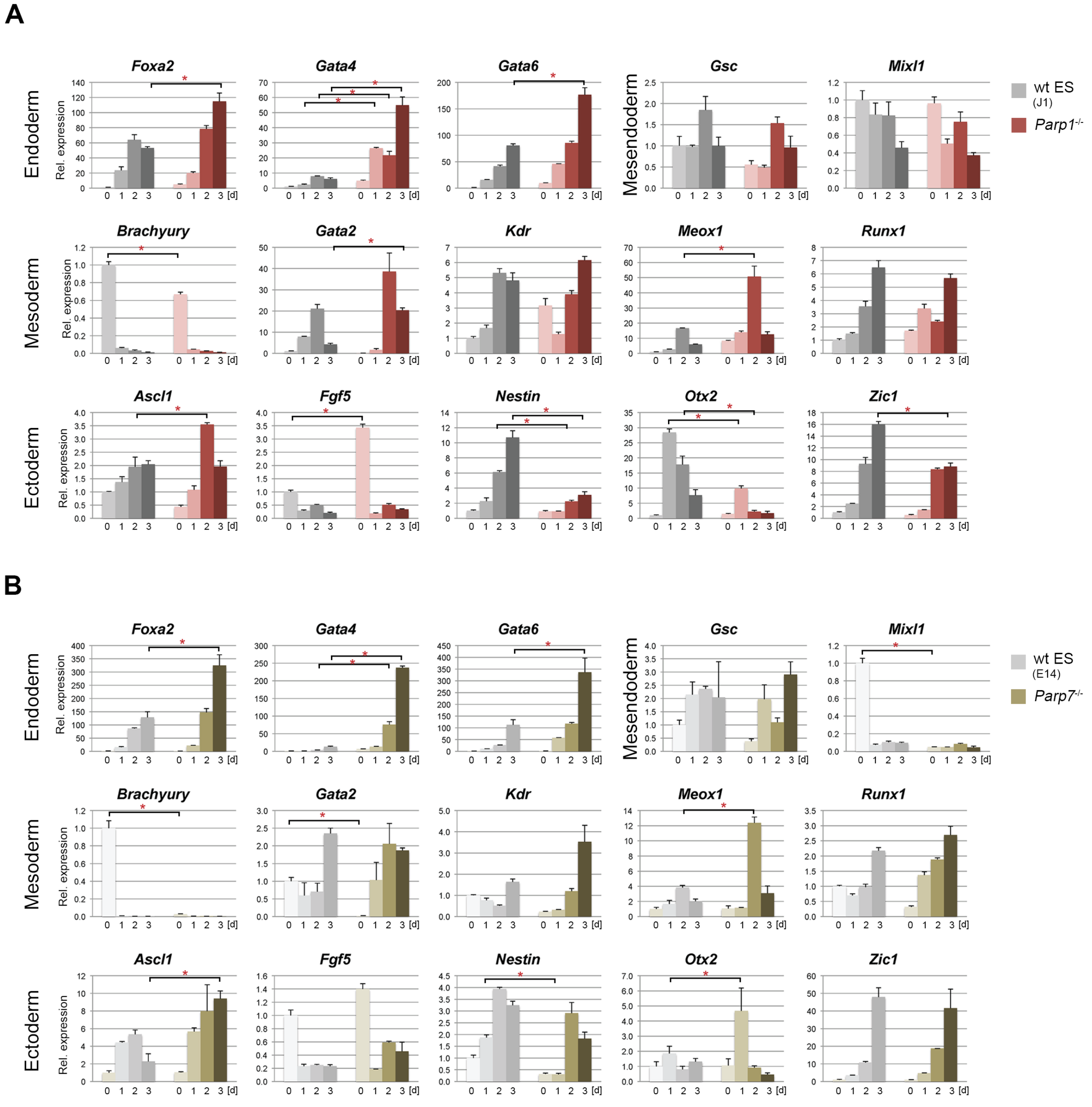

Supplementary Figure S2. Progression of differentiation of *Parp1*$^{-/-}$ (**A**) and *Parp7*$^{-/-}$ (**B**) ES cells upon exposure to retinoic acid for 1, 2, and 3 days. Marker analysis by RT-qPCR as in Figure 3.

Supplementary Figure S3

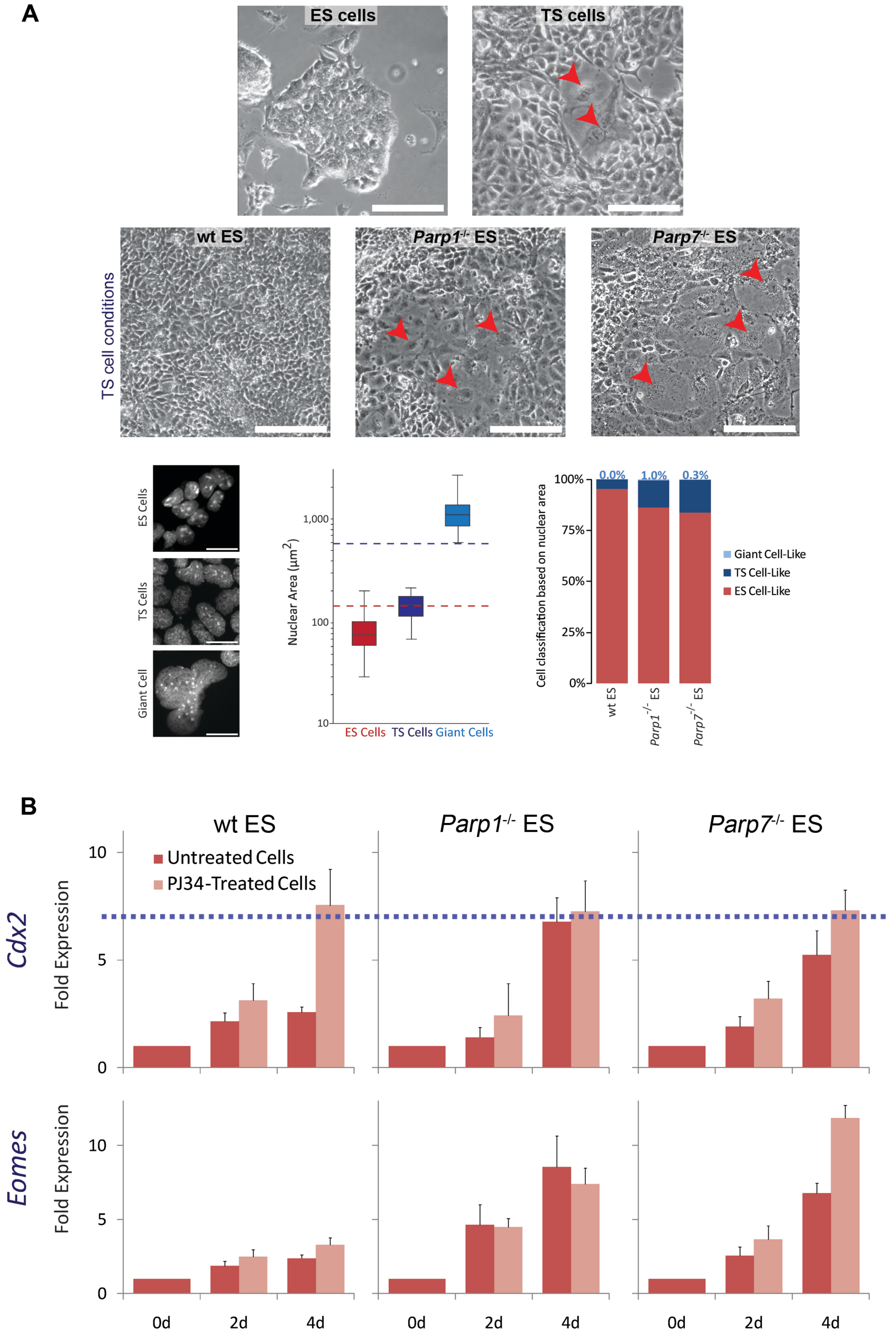

Supplementary Figure S3. Morphological and gene expression changes associated with loss of Parp1 and Parp7, or inhibition of PARylating activity. (**A**) Phase contrast images of ES and TS cells cultured in their respective standard conditions, as well as of wildtype (wt), *Parp1*$^{-/-}$ and *Parp7*$^{-/-}$ ES cells cultured in TS cell conditions. Arrowheads indicate nuclei of trophoblast giant cells (TS) or enlarged, giant cell-like cells (*Parp1*$^{-/-}$ and *Parp7*$^{-/-}$). Area measurements of DAPI-stained nuclei of ES, TS and trophoblast giant cells were used to calculate the proportion of *Parp1*$^{-/-}$ and *Parp7*$^{-/-}$ ES cells with trophoblast-like appearance. Scale bars: 200µm. (**B**) RT-qPCR expression analysis of *Cdx2* and *Eomes* in ES cell treated for 4 days with the Parp inhibitor PJ34, compared to *Parp1*- and *Parp7*-deficient ES cells. *Cdx2* becomes up-regulated to levels similar to those observed in the mutant ES cell lines by chemical Parp inhibition alone, indicated by the dotted line. No further increase in *Cdx2* expression is observed in *Parp1*- (and *Parp7*-) deficient ES cells upon Parp inhibition.

Supplementary Figure S4

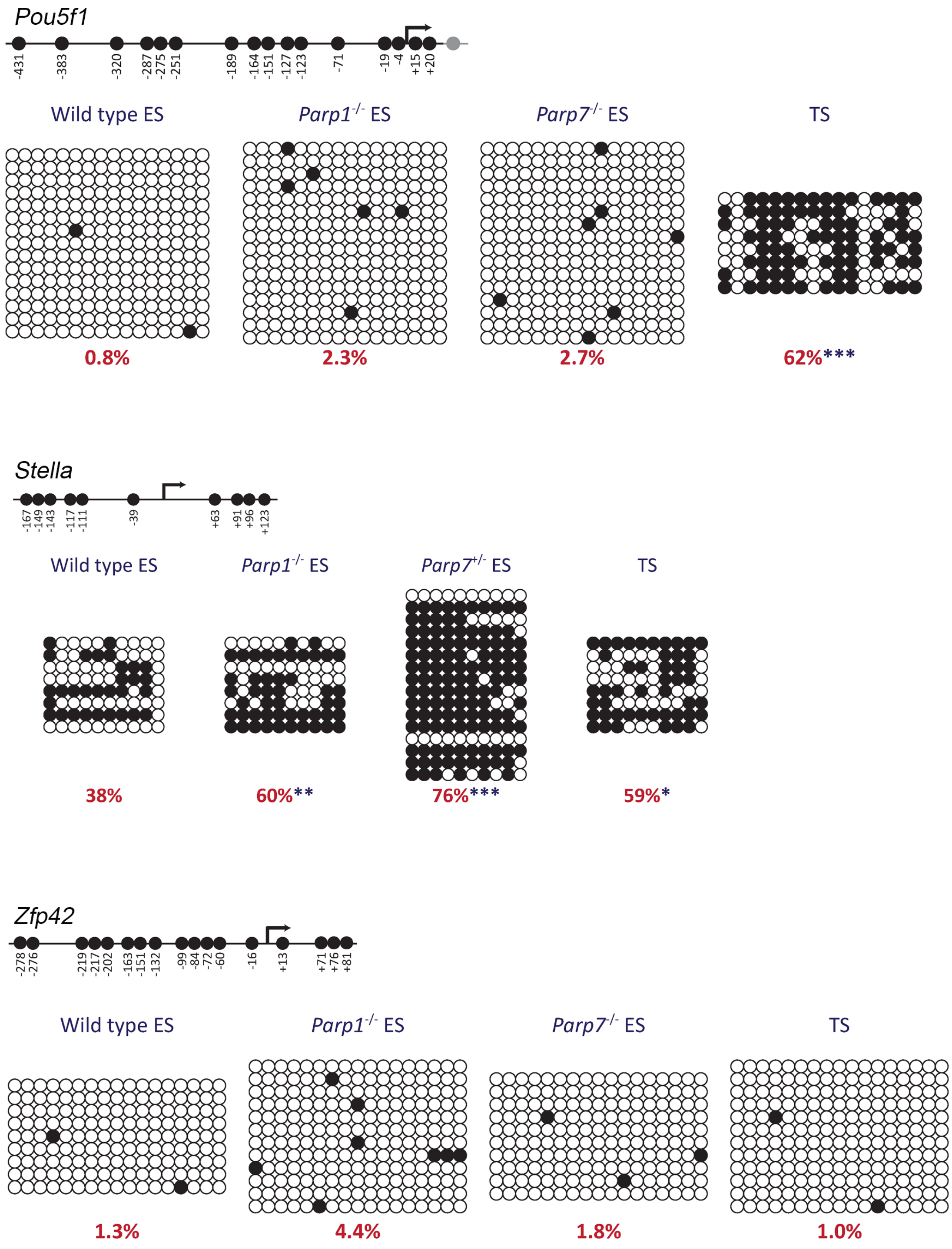

Supplementary Figure S4. Bisulphite sequencing data of wildtype (wt), *Parp1*$^{-/-}$ and *Parp7*$^{-/-}$ ES cells for the *Pou5f1*, *Stella* and *Zfp42* loci, as indicated.

Supplementary Figure S5

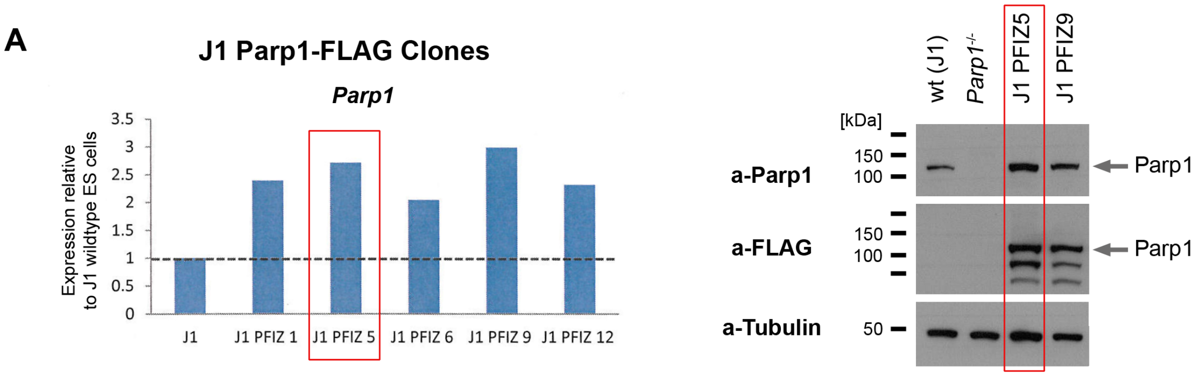

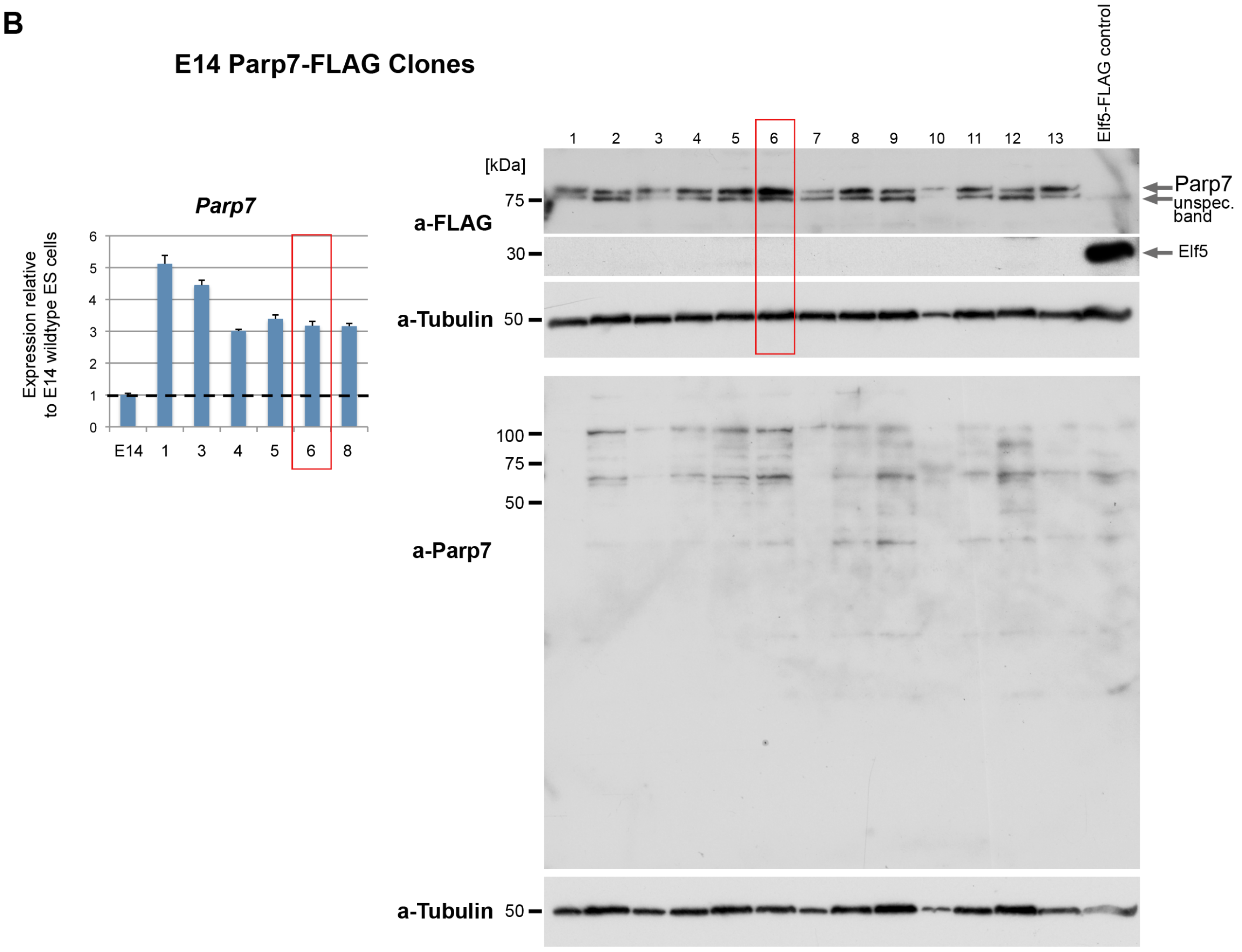

Supplementary Figure S5. Characterization of stable cell lines. (**A**) A C-terminally FLAG-tagged *Parp1* construct was introduced into J1 wildtype ES cells and stably transfected clones picked and expanded. Analysis was by RT-qPCR and by Western blotting against FLAG and endogenous Parp1 protein. The selected clone, highlighted, expresses approximately 1-2x levels of FLAG-tagged to endogenous Parp1 (thus resulting in 2-3x total *Parp1* expression levels by RT-qPCR with common primers). Tubulin served as loading control. (**B**) Equivalent analysis for E14 wt ES cell clones stably transfected with a *Parp7*-FLAG expression construct. A FLAG-tagged *Elf5* expression construct was used as Western blot control. The anti-FLAG Western blot detects the expected ~76 kDa Parp7 band in all ES cell clones. The Parp7 antibody was of low specificity, as was also evident by immunofluorescence (Figure 1E).

Supplementary Figure S6

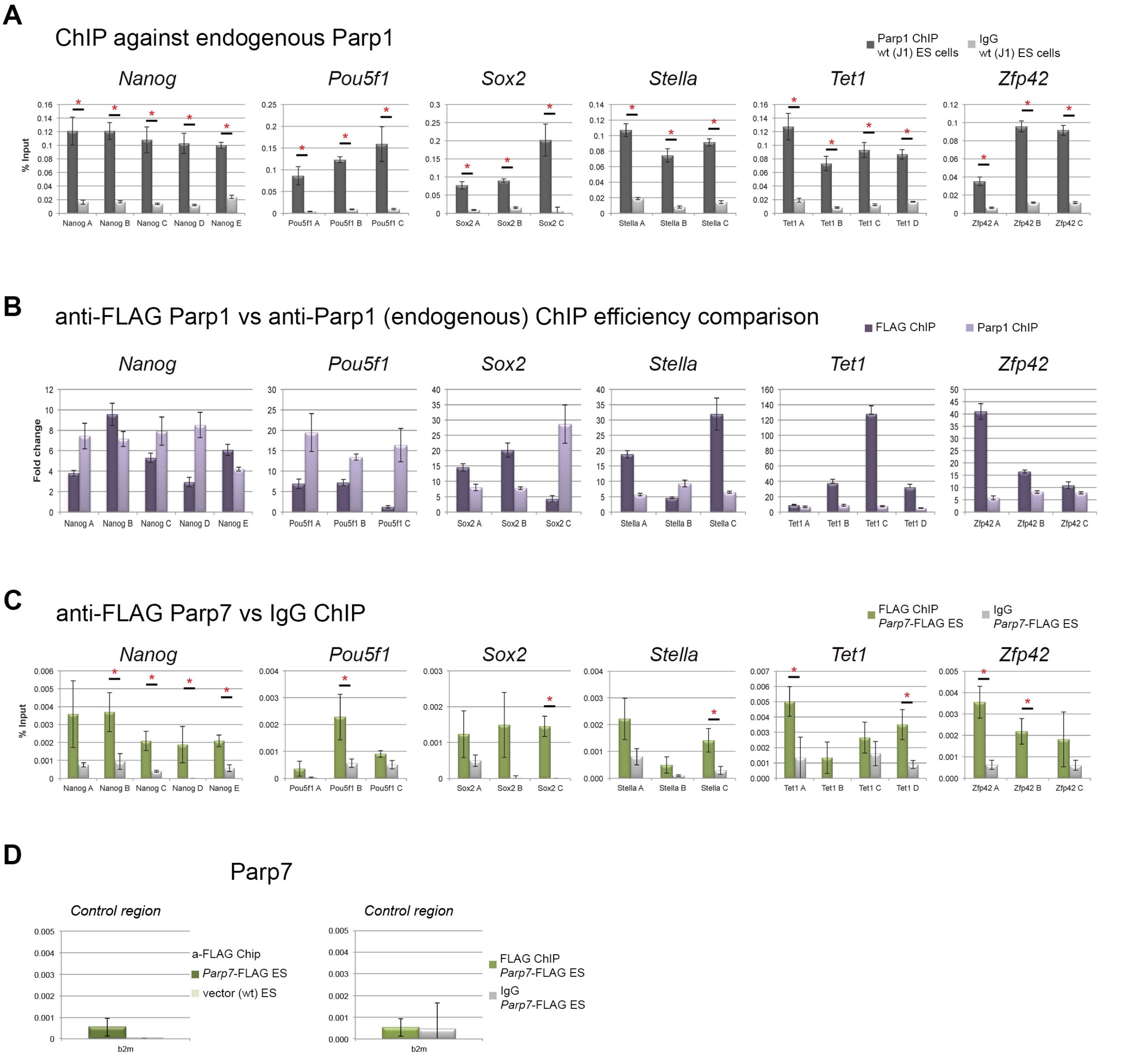

Supplementary Figure S6. Parp1 and Parp7 occupancy of pluripotency loci. (**A**) Chromatin immunoprecipitation (ChIP) against endogenous Parp1 protein in wildtype ES cells showing broad occupancy to all regions analzyed, corroborating the results obtained using ChIP against the FLAG-tagged version (Figure 6C). (**B**) Comparison of Parp1 ChIP efficiency between the anti-FLAG and anti-Parp1 antibody. Generally, the anti-FLAG ChIP is more efficient and more reliable. (**C**) Anti-FLAG ChIP against Parp7 on a wildtype ES cell line (No. 6, Supplementary Figure S5B) stably expressing a C-terminally tagged Parp7-FLAG construct, controlled for by using isotype-matched IgG on the same cell line. These data are an additional control to those in Figure 6C where the control is an anti-FLAG ChIP on “empty” wildtype (E14) ES cells. (**D**) Analysis of a control genomic locus (b2m) shows no Parp7 enrichment.

Primers used:

Pluripotency markers

**Klf4**	GAGTTCCTCACGCCAACG	CGGGAAGGGAGAAGACACT
**Klf5**	CCGGAGACGATCTGAAACAC	CAGATACTTCTCCATTTCACATCTTG
**Nanog**	TACCTCAGCCTCCAGCAGATG	CCAGATGCGTTCACCAGATAG
**Pecam1**	CCAACAGAGCCAGCAGTATGAGG	TGACAACCACCGCAATGAGC
**Pou5f1**	GAAGCCGACAACAATGAGAACC	CTCCAGACTCCACCTCACACG
**Sox2**	GAGTGGAAACTTTTGTCCGAGA	GAAGCGTGTACTTATCCTTCTTCAT
**Stella**	AGACTTGTTCGGATTGAGCAGAGAC	CCAGGGCAGCGTACAATGTAAG
**Tbx3**	GAACCTACCTGTTCCCGGAAA	CCATTGCCAGTGTCTCGAAAAC
**Tet1**	GAGCCTGTTCCTCGATGTGG	CAAACCCACCTGAGGCTGTT
**Tet2**	AACCTGGCTACTGTCATTGCTCCA	ATGTTCTGCTGGTCTCTGTGGGAA
**Tet3**	TCCGGATTGAGAAGGTCATC	CCAGGCCAGGATCAAGATAA
**Zfp42 (Rex1)**	ATCTTACCTGCCTCTGCCTCCTG	GCTTCCTTCTTGAACAATGCCTATG
**Dnmt3b**	TGGTGATTGGTGGAAGCC	AATGGACGGTTGTCGCC
**Prdm14**	kind gift of the Reik lab, Ficz et al 2013	

EB differentiation markers

**Ascl1**	CGGTCTCGTCCTACTCCTCC	ATCTGCTGCCATCCTGCTTC
**Brachyury**	TCATAACGCCAGCCCACCTAC	GCGTCAGTGGTGTGTAATGTGC
**Fgf5**	TGTGTCTCAGGGGATTGTAGG	AGCTGTTTTCTTGGAATCTCTCC
**Foxa2**	GGAGCCGTGAAGATGGAAG	TGTGTTCATGCCATTCATCC
**Gata2**	CTACCACAAGATGAATGGACAGAA	CTTCTCTTGCATGCACTTGGAG
**Gata4**	TAGCAGCAGCAGCAGCAGTG	GCATAGCCTTGTGGGGACAG
**Gata6**	TCTACACAAGCGACCACCTCAG	GCCAGAGCACACCAAGAATCC
**Gsc**	ACCGCACCATCTTCACCG	CGCTTCTGTCGTCTCCACTTG
**Kdr**	TTTGGCAAATACAACCCTTCAGA	GCAGAAGATACTGTCACCACC
**Meox1**	GACAGCAGCATACCCCGAC	CGTTGAAGATTCGCTCAGTCC
**Mixl1**	GTCTTCCGACAGACCATGTACC	CCCGCCTTGAGGATAAGGG
**Otx2**	GCTCTGTTTGCCAAGACCCG	TGGCGGCACTTAGCTCTTCG
**Runx1**	CGCCACAAGTTGCCACCTAC	GCTCCAATTCACTGAGCCGC
**Sox1**	Qiagen QT00289338	
**Zic1**	TGCGATAAGTCCTACACGCAC	CGTGGACGACTCATACCCC

ChIP primers

**Nanog –F1**	CCACGGTGAAGGTGCCACATC
**Nanog –R2**	TCTTTCCCTCTCTCCCAAGTGCTG
**Nanog –F2**	AGTCAGGCTGGGCAATGGAG
**Nanog –R2**	TGCCATCACTGCCACTACGAC
**Nanog –F3**	TTAAATTGGGCATGGTGGTAGAC
**Nanog –R3**	TTCTTGCTTGCTCTTCACATTGG
**Nanog –F4**	TCCCCAGGTTTCCCAATGTG
**Nanog –R4**	TATTCATCTTTTAACCACGGCTGC
**Pou5f1 –F1**	GGGTGTGGGGAGGTTGTAGC
**Pou5f1 –R1**	AGCCGCCAAGTTCACAAAGC
**Pou5f1 –F2**	AACGCAGAGCCAGCACTTCTC
**Pou5f1 –R2**	AACCCAGTATTTCAGCCCATGTC
**Pou5f1 –F3**	TTTGTGGTGCGATGGGGC
**Pou5f1 –R3**	GTTGGGGACGTCTGGACAGG
**Pou5f1 –F4**	GTGCCCCTGCCGTTCTATTG
**Pou5f1 –R4**	CTCTGGAGGTGAGAAGCCTTGAG
**Sox2 –F1**	AGATGTGGGGGTGGGGAAGAG
**Sox2 –R1**	CACGCTCCGCTCATTGTCCTTAC
**Sox2 –F2**	GTTCCTTGAGCCACACCTGAGTC
**Sox2 –R2**	CCTGGCTTCCGTGTCATCG
**Sox2 –F3**	GGATAACTGTTCAGCCACCAAGAT
**Sox2 –R3**	TTATTCACGCTGCTCAGGGAAG
**Stella –F1**	ATCTCAGGGGTGTCCGTTGC
**Stella –R1**	TGAGCCTTAATAGTATGGGACTTGCC
**Stella –F2**	CGAGAGCGGGGAATCCTACAG
**Stella –R2**	CCGAGTCTACCCCCAGGCTAC
**Stella –F3**	CCGTACCTGTGGAGAACAAGAGTG
**Stella –R3**	CCGTTGAGGCTGGAGTTGC
**Tet1 –F1**	GCGCATTGTTGCTTTGGCTG
**Tet1 –R2**	GCAGGTCTTACCTGTCTAGAACTCCA
**Tet1 –F2**	TCCTTCTGCCCTGCCTCAGT
**Tet1 –R2**	GCCACCTCAACAAGAGGCGT
**Tet1 –F3**	CTTTGGAAGAGCAGTCGGGGG
**Tet1 –R3**	ATGGCGCACTCCTCCTTTGA
**Tet1 –F4**	CCCCAACTCCCACCAAACCC
**Tet1 –R4**	AAACCCTTGTGGGACGGCTG
**Zfp42 –F1**	GTGTGTGAGTGATTTGCCTGTATGTATG
**Zfp42 –R1**	ATTTCTGCTTTTCTGAAGACCTGGG
**Zfp42 –F2**	GCATGACCCACGCTCTCAAAG
**Zfp42 –R2**	GGACCCCGCTACAAAGTACACTAG
**Zfp42 –F3**	AGATAGTCTTCTTCTCTCAATAGAGTGAGTGTG
**Zfp42 –R3**	ATTCCCCAGCCTGCGTTACA

Sequenom and Bisulphite primers

**Elf5**	Outside F	GTGGAAAGGTTAGTGAAAGTATTG
	Inside F	AGGAAGAGAGTGATTTTTTTTTTGTTTTTTGAT
	Inside R	CAGTAATACGACTCACTATGGGAGAAGGCTCCTAATATCTATTCATTACAACCT
	Outside R	AAAAAATTCAAACCTAATATCTA
**Nanog**	Outside F	TATTTTTGGAGGGAAGATTTTTTTAGG
	Inside F	AGGAAGAGAGGGTTTTTTAATGTGAAGAGTAAGTAAG
	Inside R	CAGTAATACGACTCACTATGGGAGAAGGCTCCAACCAAATCAACCTATCTAAAAA
	Outside R	CAACCTTCCCACAAAAAAAACAAAAC
**Pou5f1**	Outside F	GTGGTTTTAGAAATAATTGGTATA
	Inside F	AGGAAGAGAGGGTTTTTTAGAGGATGGTTGAGTG
	Inside R	CAGTAATACGACTCACTATGGGAGAAGGCTTCCAACCCTACTAACCCATCACC
	Outside R	TAAAAACCCTTAAAAACTTAACC
**Sox2**	Outside F	TTATTTT**T**GGTTTTGTTAGTTTTTTG
	Inside F	AGGAAGAGAGATTATTTATGGGTTTTGTTTTATTTTGG
	Inside R	CAGTAATACGACTCACTATGGGAGAAGGCTTAATAAACAACCATCCATATAATAAAAAC
	Outside R	TTACAAACACTCTCTTCTCTACCTTA
**Stella**	Outside F	ATTTTGTGATTAGGGTTGGTTTAGAA
	Inside F	AGGAAGAGAGTTTTTGGAATTGGTTGGGATTG
	Inside R	CAGTAATACGACTCACTATGGGAGAAGGCTCTTCTAAAAAATTTCAAAATCCTTCATT
	Outside R	CCAAAACATCCTCTTCATCTTTCTTCT
**Zfp42**	Outside F	GTTATAAGTTTGTAATAGAGGTATTG
	Inside F	AGGAAGAGAGGATGTGATTGAGTTTTAAGGTTAGG
	Inside R	CAGTAATACGACTCACTATGGGAGAAGGCTAAACAAAACTTACCTAATACCCACAAC
	Outside R	AAAAACAAATCCCAACCATTTCATAAAAC
**Elf5**	F1	GTGGAAAGGTTAGTGAAAGTATTG
	R1	AAAAAATTCAAACCTAATATCTA

	F2	TGATTTTTTTTTTGTTTTTTGAT
	R2	CCTAATATCTATTCATTACAACCT
**Nanog**	F1	TATTTTTGGAGGGAAGATTTTTTTAGG
	R1	CAACCTTCCCACAAAAAAAACAAAAC
	F2	GGTTTTTTAATGTGAAGAGTAAGTAAG
	R2	CCAACCAAATCAACCTATCTAAAAA
**Pou5f1**	F1	TGGGTTGAAATATTGGGTTTATTT
	R1	CTAAAACCAAATATCCAACCATA
	F2	GGGTTTATTTATATTTAGGATTTTAGA
	R2	ATATCCAACCATAAAAAAAATAAACACCC
**Sox2**	F1	TTATTTT**T**GGTTTTGTTAGTTTTTTG
	R1	TTACAAACACTCTCTTCTCTACCTTA
	F2	ATTATTTATGGGTTTTGTTTTATTTTGG
	R2	TAATAAACAACCATCCATATAATAAAAAC
**Stella**	F1	ATTTTGTGATTAGGGTTGGTTTAGAA
	R1	CCAAAACATCCTCTTCATCTTTCTTCT
	F2	TTTTTGGAATTGGTTGGGATTG
	R2	CTTCTAAAAAATTTCAAAATCCTTCATT
**Zfp42**	F1 (A)	GGGGATGATAGGAGGTTTATTTTATT
	R1 (A)	AAATACAATTTCTACCTACCTACCTAC
	F2 (A)	GAGGAATGAGAGATATTTTATAAATAG
	R2 (A)	CACACCGAAAACTTTCTTTACACTTTC
	F1 (B)	GTTATAAGTTTGTAATAGAGGTATTG
	R1 (B)	AAAAACAAATCCCAACCATTTCATAAAAC
	F2 (B)	GATGTGATTGAGTTTTAAGGTTAGG
	R2 (B)	AAACAAAACTTACCTAATACCCACAAC
